# Differences in Injury Incidence Between Player Positions Across All Rugby Formats—A Systematic Review and Meta‐Analysis

**DOI:** 10.1111/sms.70102

**Published:** 2025-07-07

**Authors:** Matias Vaajala, Juho Laaksonen, Rasmus Liukkonen, Oskari Pakarinen, Ilari Kuitunen

**Affiliations:** ^1^ Faculty of Medicine and Health Technology Tampere University Tampere Finland; ^2^ Department of Surgery Central Finland Central Hospital Jyväskylä Finland; ^3^ Department of Surgery Päijät‐Häme Central Hospital Lahti Finland; ^4^ Department of Pediatrics Kuopio University Hospital Kuopio Finland; ^5^ Institute of Clinical Medicine University of Eastern Finland Kuopio Finland

**Keywords:** epidemiology, incidence, prevention, Rugby

## Abstract

Rugby is a high‐intensity contact sport with a high incidence of injuries. The aim of this systematic review and meta‐analysis was to compare the injury incidences between forwards and backs in all rugby formats (Rugby Union, Seven, and League). The PubMed (National Library of Medicine), Web of Science (Clarivate), Scopus (Elsevier), and SPORTDiscus (EBSCO) databases were searched from inception to October 10, 2024. A study was eligible if the number of rugby injuries per exposure time for backs and forwards was reported. Incidence rate ratios (IRRs) with 95% confidence intervals (CI) were compared between forwards and backs. After screening 1514 studies, a total of 49 studies were included. The total exposure time was 902 280 player‐hours for backs and 1 127 610 player‐hours for forwards. Forwards had a slightly higher incidence of injuries in Rugby Union (IRR 1.03, CI 1.00–1.06) and Rugby League (IRR 1.31, CI 1.23–1.41). However, forwards had a lower overall incidence of injuries in Rugby Sevens (pooled IRR 0.78, CI 0.73–0.85). Strain injuries and injuries caused by tackling, running, or collision were more common in backs across rugby formats. In addition to a more standardized definition of injuries and more comprehensive subgroup analyses in future studies, different trends and patterns in injury occurrence regarding playing positions in all rugby formats should be further studied to develop more targeted injury prevention strategies in forwards and backs.

**Trial Registration:** The study protocol was prospectively registered in the PROSPERO database (CRD42025645250)

## 1 Introduction

Rugby League and Rugby Union are two of the most physically demanding contact sports, played with 13 or 15 players per team [1, 2]. Rugby Sevens, a variant of Rugby Union, features teams of seven players and is known for its fast‐paced nature and shorter match duration [3]. According to the World Rugby international federation, the total number of registered players is over 8 million in over 120 countries worldwide [4]. All three of the rugby formats require intense physicality, including frequent collisions, high‐speed tackles, and prolonged physical engagement [5].

In recent years, concerns have been raised about the high incidence of injuries in all three rugby formats, particularly regarding concussions and their long‐term effects on player health [6]. Previous systematic reviews have reported high incidence rates in all formats. According to Yeomans et al., the incidence of injuries in male Rugby Union was 47 injuries per 1000 playing‐hours [7]. Williams et al. reported an incidence of 91 per 1000 player‐hours in professional Rugby Union [8]. King et al. investigated the match and training injuries in Rugby League, which were reported to be 89 and 12 per 1000 player‐hours for match and training injuries [9].

Rugby players can generally be categorized into forwards and backs, with distinct physical and tactical roles. Forwards are typically involved in physical contests such as scrums and rucks in Rugby Union and high‐impact tackles in Rugby League [10]. They are generally taller, heavier, and possess greater strength and muscular endurance suited to sustained physical engagements, such as repeatedly moving from ruck to ruck [11, 12]. In contrast, backs rely more on speed, agility, and repeated sprint ability, performing actions like consecutive high‐speed runs during open play [11, 12]. These positional differences influence exposure to different types of impacts and injury risks. Studies have shown that forwards tend to sustain more contact‐related injuries, whereas backs experience more sprint‐related muscle injuries [13]. King et al. found that the hooker playing position continues to have the highest match injury risk with an incidence of approximately 93 per 1000 match‐hours [9].

While numerous studies have investigated injury rates in Rugby Union, many have already identified clear differences in injury incidence between forwards and backs. However, given the evolving nature of the sport and ongoing changes in player roles, rules, and training practices, there is value in synthesizing recent findings to provide an up‐to‐date, comprehensive evaluation. Moreover, while Rugby Union has been relatively well studied, there remains a lack of research specifically examining positional differences in Rugby League and Rugby Sevens. This systematic review and meta‐analysis aimed to provide a comprehensive evaluation of injury incidence in Rugby Union, Rugby League, and Rugby Sevens, with a specific focus on positional differences.

## 2 Materials and Methods

### 2.1 Information Sources and Search Strategy

The databases PubMed (National Library of Medicine), Web of Science (Clarivate), Scopus (Elsevier), and SPORTDiscus (EBSCO) were searched from their inception until October 10, 2024, using the following search terms: “Rugby AND injury AND (epidemiology or incidence).” No filters or language restrictions were used in the search. The review was reported according to the Preferred Reporting Items for Systematic Reviews and Meta‐Analyses (PRISMA) 2020 checklist and PERSiST guidance (PRISMA in Exercise, Rehabilitation, Sport medicine, and Sports science). The study protocol was prospectively registered in the PROSPERO database (CRD42025645250).

### 2.2 Eligibility Criteria and Selection Process

The search results were imported into Covidence software (Veritas Healthcare, Melbourne, Australia) for screening, and duplicate entries were eliminated. All five authors participated in the study screening process. Two authors independently screened all returns at both the abstract and full‐text screening stages. Any disagreements were resolved by discussion and achieving mutual consensus. During the abstract screening process, any study possibly reporting rugby injuries, despite the position‐stratified requirement, was included. All study designs (e.g., retrospective and prospective study) and all injury types (e.g., medical attention, time‐loss, and anatomically specific injuries) were included during the screening process. During the full‐text phase, the study was eligible for our analysis if the number of injuries and exposure times were reported by playing positions (forwards or backs). A study was excluded if the full text was not available or the English version was not available. Furthermore, if the study included a cohort used in other studies, we included the most recent one.

Our study utilized the basic stratification commonly used in rugby to divide the players in forwards (Rugby‐15s: loose‐head prop, hooker, tight‐head prop, second row, blindside flanker, open side flanker, number 8; Rugby‐7s: loose‐head prop, hooker, tight‐head prop) and backs (Rugby‐15s: left wing, scrum half, fly half, inside center, outside center, fullback; Rugby‐7s: scrum half, fly half, center, wing) [10].

### 2.3 Data Extraction

Data were extracted and recorded in an Excel spreadsheet. All authors participated in data extraction. One of the authors extracted, and another author validated the extracted data to reduce potential extraction errors. The extracted information from each study included author names, journal, study title, year of publication, country of the research, study duration, design, participant details (sex, playing level, youth/adult), and injury definition. We had no criteria regarding the classification (e.g., time‐loss, medical attention, any physical pain) of an injury and used the classifications that the original studies had used. The following data about the participants and injuries was collected separately for forwards and backs: number of participants, anthropometric data for the participants (age, stature, mass, BMI), total number of injuries, exposure time, and exposure units.

### 2.4 Risk of Bias Assessment

The Joanna Briggs Institute (JBI) critical appraisal list for prevalence studies was used to assess the risk of bias [14]. The Risk of Bias (RoB) assessment was conducted independently by two authors (M.V. and J.L.) and conflicts were resolved through mutual consensus. The evaluation covered nine domains, and each domain was rated as “yes” or “no.”

### 2.5 Outcome Measures

The primary outcome was the incidence rate ratios (IRRs) with 95% confidence intervals (CI) between forwards and backs in Rugby Union, Rugby Sevens, and Rugby League. The IRRs between forwards and backs were also calculated separately for practices and games, for anatomical locations (head/neck, upper limb, trunk/back and lower limb), for injury types (concussion, strain, sprain, skin injuries, other nerve injuries, and bone injuries), and cause of injury (tackles, rucks or mauls, running, collisions) as reported by original authors. The IRRs were calculated based on the given information (number of injuries, and the total exposure time separately for backs and forwards) in the original studies. In addition, the pooled overall injury incidence in all Rugby formats was reported for forwards and backs, expressed as the number of injuries per 1000 player‐hours. This was determined for each study by dividing the total number of injuries by the total number of player‐hours, which included participation in both games and practices.

### 2.6 Statistics

Statistical analyses were performed in accordance with the guidelines provided in the latest edition of the Cochrane Handbook for Systematic Reviews of Interventions [15]. Injury incidence was calculated separately for each study, and pooled incidences were reported with 95% confidence intervals (CIs) using a random‐effects model. IRRs with 95% CIs were compared between backs and forwards. Random‐effects models were applied due to the unavoidable heterogeneity in player‐specific factors (e.g., injury history, level of play, physical attributes). Study heterogeneity was assessed using the *I* ^2^ statistic. For studies reporting both injury incidence and the number of injuries, exposure time was calculated based on the available data. As the subgroup analyses, IRRs were conducted to reduce anticipated heterogeneity, focusing on practice versus game settings, professional versus amateur, injury location, injury type, and cause of injury. Forest plots were used for visual presentation of the analyses. In addition, sensitivity analyses for medical attention and time‐loss injuries were performed. A funnel plot created by plotting IRRs against their standard errors was utilized to examine the potential for publication bias. All analyses were performed using the *meta* package from R (version 4.1.2, R Foundation for Statistical Computing, Vienna, Austria) [16, 17].

## 3 Results

**FIGURE 1:**
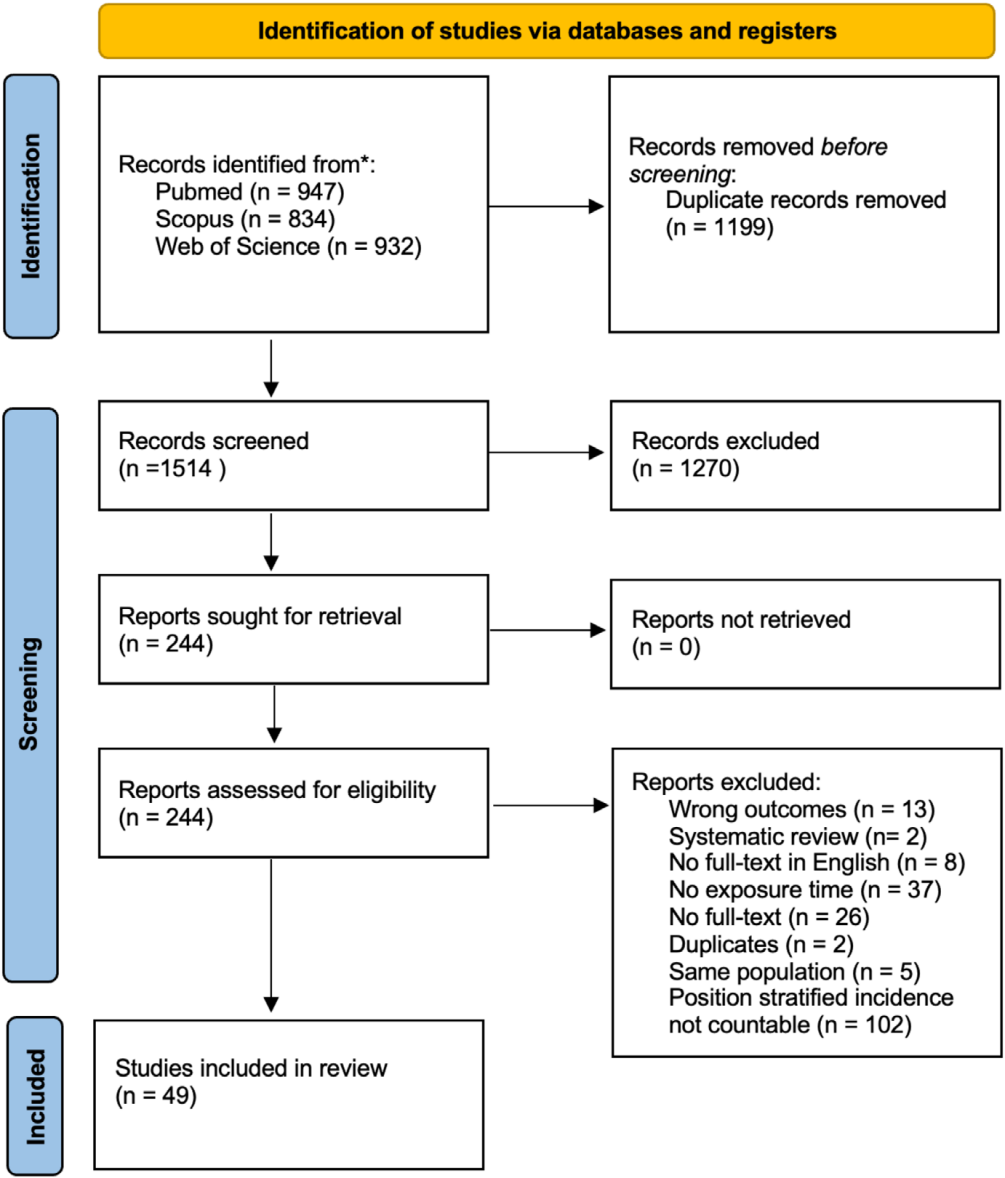
PRISMA flowchart of the study selection process.

In total, 2713 articles were found from the literature search. Of these, a total of 1199 were duplicates. A total of 1514 abstracts were screened, leading to 244 studies in the full‐text phase. Of these, a total of 49 studies passed the eligibility assessment and were included in the analyses (Figure 1) [18, 19, 20, 21, 22, 23, 24, 25, 26, 27, 28, 29, 30, 31, 32, 33, 34, 35, 36, 37, 38, 39, 40, 41, 42, 43, 44, 45, 46, 47, 48, 49, 50, 51, 52, 53, 54, 55, 56, 57, 58, 59, 60, 61, 62, 63, 64, 65, 66]. A total of five studies had a population consisting of Rugby League players [40, 41, 45, 61, 64]; 7 out of the remaining 44 studies focused on Rugby Sevens [30, 33, 38, 39, 49, 50, 51], while the remaining 37 were Rugby Union. In total, five studies reported injuries only for females [39, 46, 51, 57, 60], while the cohort of three studies included both sexes [18, 49, 50]. A total of six studies included only youth participants [42, 47, 50, 52, 55, 64], and two studies included both youth and adults [51, 67], while the 41 remaining studies included only adults. In total, 13 studies included amateur players [42, 45, 46, 47, 49, 50, 52, 54, 55, 62, 63, 64, 65, 67], three studies included semi‐professional players [26, 40, 60], one study included players from all playing levels [33], and the remaining 43 studies included professional players. The majority of the included studies were conducted in Europe (*n* = 23, 46%) (Table S1).

### 3.1 Risk of Bias Assessment

RoB‐assessment was conducted for each included study. The quality of included studies was high. A total of 41 out of the included 49 studies received high (7 items or more properly addressed) as the overall quality, and eight studies received moderate (5 or 6 items properly addressed). The minimum number of properly addressed items was 5, which was addressed by three studies. Three studies addressed 6 items properly. None of the studies were excluded due to high RoB. The complete RoB‐assessment template is provided as Supporting Information (see Table S2).

### 3.2 Participant Characteristics

Anthropometric data, including the age, height, and mass, was reported in 25 out of the 49 studies included. The mean age of the participants ranged between 17.3 and 29.2 years for backs, and between 17.3 and 29.0 for forwards in the included studies. Among the studies including anthropometric description about age, height, and mass, forwards were notably taller (mean height difference + 5.0 cm, 95% CI 4.02–6.48) and heavier (mean mass difference + 14.82 kg, 95% CI 12.4–17.60) (Table S3).

### 3.3 The Pooled Incidence and Incidence Rate Ratios of Rugby Injuries

**FIGURE 2:**
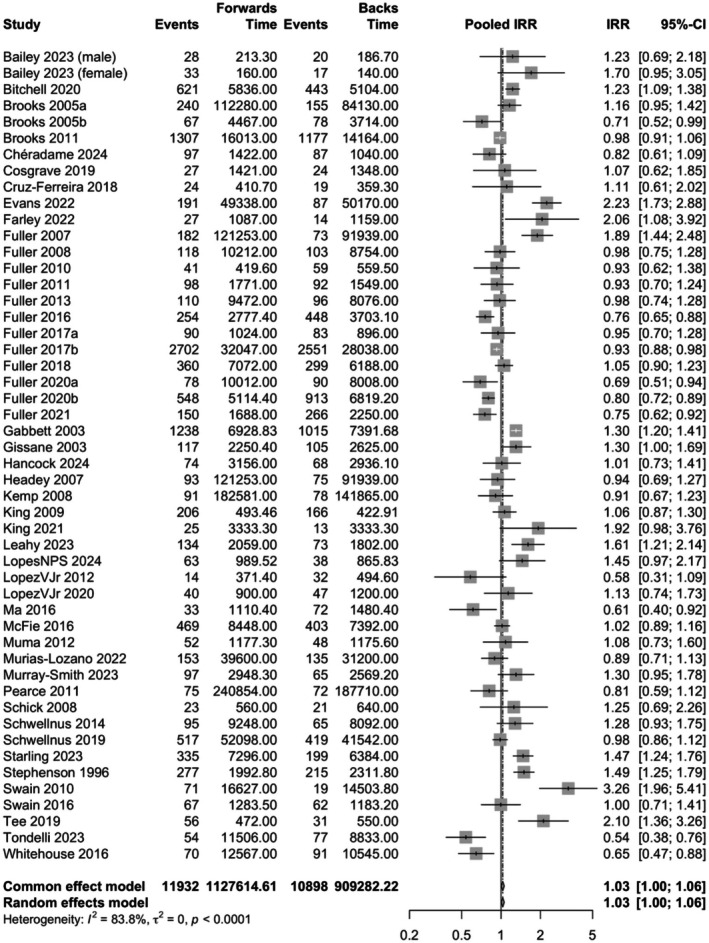
Incidence rate ratios (IRRs) with 95% confidence intervals (CI) for overall injury incidence between backs and forwards. Points located to the right of the vertical line suggest that the incidence of injuries is higher among forwards compared to backs.

The total exposure time for backs was 902 280 player‐hours (training = 731 433 h, match = 163 391 h, unknown combination of these = 7456 h), and a total of 1 127 610 player‐hours (training = 933 655 h, match = 177 502 h, unknown combination of these = 16 453 h) for forwards. During this time, backs suffered a total of 10 898 injuries, and forwards suffered a total of 11 932 injuries. The pooled overall incidence of all rugby injuries for backs was 23.1 (CI 14.7–36.4, *I* ^2^ = 99.7%) injuries per 1000 player‐hours, and 25.3 (CI 16.2–39.6, *I* ^2^ = 99.8%) injuries per 1000 player‐hours for forwards (Figures S1 and S2) Forwards had a slightly higher incidence of injuries when compared to backs (pooled IRR 1.03, CI 1.00–1.06, *I* ^2^ = 83.8%) (Figure 2).

### 3.4 Rugby Union

**FIGURE 3:**
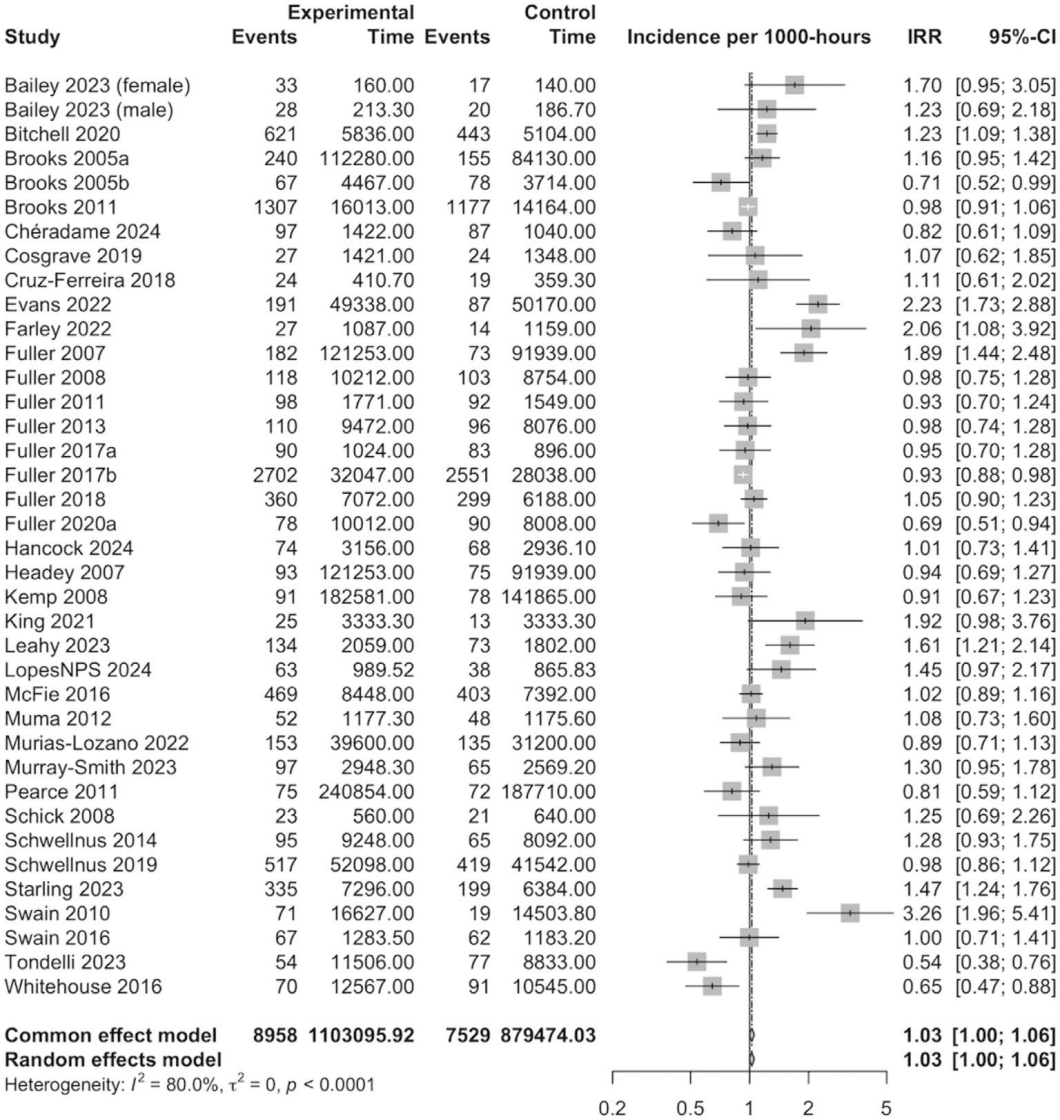
Incidence rate ratios (IRRs) with 95% confidence intervals (CI) for overall injuries between backs and forwards in rugby union. Points located to the right of the vertical line suggest that the incidence of injuries is higher among forwards compared to backs.

A total of 37 studies investigated the incidence of injuries in Rugby Union. Forwards had a higher incidence of injuries when compared to backs (pooled IRR 1.03, CI 1.00–1.06, study *n* = 37, *I* ^2^ = 80.0%) (Figure 3). No evidence of difference was found in the incidence of training injuries (pooled IRR 0.95, CI 0.85–1.06, study *n* = 15, *I* ^2^ = 67.6%), and match injuries (pooled IRR 1.02, CI 0.99–1.06, study *n* = 34, *I* ^2^ = 67.5%) between forwards and backs in Rugby Union. Also, no evidence of difference was found in the incidence of overall injuries between professional (pooled IRR 0.99, CI 0.96–1.02, study *n* = 25, *I* ^2^ = 68.0%) and amateur (pooled IRR 1.08, CI 0.99–1.18, study *n* = 10, *I* ^2^ = 81.6%) forwards and backs (Figures S3–S6). Only one study reported the incidence between forwards and backs among female players, and no studies reported injuries in youth, and therefore no data synthesis was performed.

A total of 16 studies out of the 37 studies in Rugby Union reported injuries in different anatomic locations, types of injuries, or causes of the injury. Forwards had a higher incidence of head injuries (pooled IRR 1.15, CI 1.00–1.33, study *n* = 14, *I* ^2^ = 14.9%), arm and elbow injuries (pooled IRR 2.38, CI 1.11–5.11, study *n* = 7, *I* ^2^ = 0.0%), trunk and back injuries (pooled IRR 1.45, CI 1.17–1.79, study *n* = 13, *I* ^2^ = 21.0%), and calf injuries (pooled IRR 1.33, CI 1.05–1.67, study *n* = 2, *I* ^2^ = 4.9%) when compared to backs. On the other hand, forwards had a lower incidence of lower limb injuries (pooled IRR 0.87, CI 0.79–0.95, study *n* = 13, *I* ^2^ = 37.3%) and thigh injuries (pooled IRR 0.54, CI 0.45–0.64, study *n* = 7, *I* ^2^ = 77.4%) when compared to backs (Figures S7–S18 and Table 1).

**TABLE 1:** Incidence rate ratios (IRRs) with 95% confidence intervals (CIs) for injuries in different anatomic locations, by different nature, and cause of injury. Forwards were compared to backs. No studies about Rugby League reported the incidence of injuries by their location, nature, or cause and therefore are not included in the table.

Location	Rugby Union			Rugby Sevens		
	Study (*n*)	IRR (CI)	*I* ^2^ (%)	Study (*n*)	IRR (CI)	*I* ^2^ (%)
Location						
Head and neck	14	1.15 (1.00–1.33)	14.9	6	0.92 (0.77–1.10)	0.0
Upper limb	13	1.06 (0.91–1.22)	0.0	6	0.87 (0.73–1.03)	7.6
Shoulder	9	0.98 (0.81–1.18)	41.0	4	0.95 (0.75–1.20)	17.7
Arm and elbow	7	2.38 (1.11–5.11)	0.0	4	0.36 (0.17–0.76)	0.0
Forearm and wrist	7	1.18 (0.54–2.56)	0.0	4	0.73 (0.35–1.53)	0.0
Trunk and back	13	1.45 (1.17–1.79)	21.0	6	1.99 (1.42–2.78)	73.1
Lower limb	13	0.87 (0.79–0.95)	37.3	6	0.74 (0.66–0.81)	81.9
Hip and groin	6	0.80 (0.55–1.17)	24.5	4	0.90 (0.55–1.47)	0.0
Thigh	7	0.54 (0.45–0.64)	77.4	4	0.58 (0.46–0.72)	0.0
Calf	2	1.33 (1.05–1.67)	4.9	0	—	—
Knee	8	1.01 (0.78–1.31)	0.0	4	0.70 (0.57–0.85)	81.5
Foot and ankle	7	1.00 (0.78–1.30)	0.0	4	0.68 (0.56–0.82)	0.0
Injury nature						
Concussion	14	1.06 (0.92–1.22)	35.9	4	0.84 (0.68–1.05)	0.0
Sprain	12	1.11 (0.98–1.26)	49.9	4	0.79 (0.70–0.89)	0.0
Strain	12	0.81 (0.71–0.91)	16.3	4	0.59 (0.50–0.70)	0.0
Lacerations	11	1.36 (0.80–2.30)	0.0	3	1.22 (0.67–2.20)	0.0
Nerve	9	1.56 (1.01–2.40)	0.0	4	0.72 (0.37–1.42)	0.0
Bone	11	1.05 (0.77–1.45)	0.0	4	0.99 (0.77–1.27)	0.0
Injury cause						
Tackle	4	0.62 (0.49–0.79)	0.0	4	0.68 (0.61–0.76)	89.9
Ruck or maul	4	1.60 (0.99–2.61)	0.0	4	1.08 (0.82–1.41)	0.0
Running	4	0.53 (0.34–0.81)	27.0	4	0.42 (0.33–0.52)	46.4
Collision	4	0.66 (0.44–0.99)	9.3	4	0.92 (0.74–1.14)	0.0

Forwards had a higher incidence of nerve injuries (pooled IRR 1.56, CI 1.01–2.40, study *n* = 9, *I* ^2^ = 0.0%), and a lower incidence of strain injuries (pooled IRR 0.81, CI 0.71–0.91, study *n* = 12, *I* ^2^ = 16.3%), when compared to backs. Forwards had a lower incidence of injuries caused by tackling or being tackled (pooled IRR 0.62, CI 0.49–0.79, study *n* = 4, *I* ^2^ = 0.0%), caused by running (pooled IRR 0.53, CI 0.34–0.81, study *n* = 4, *I* ^2^ = 27.0%), and caused by collisions (pooled IRR 0.66, CI 0.44–0.99, study *n* = 4, *I* ^2^ = 0.0%), when compared to backs (Figures S19–S28 and Table 1).

### 3.5 Rugby Sevens

**FIGURE 4:**
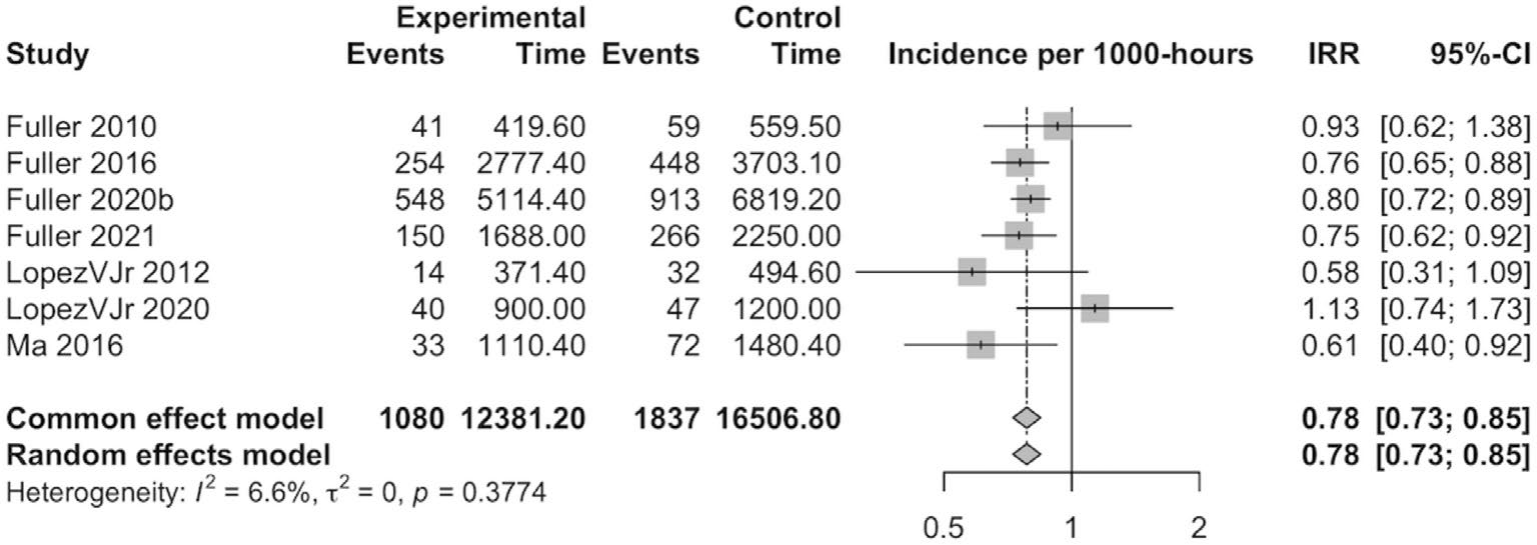
Incidence rate ratios (IRRs) with 95% confidence intervals (CI) for overall injuries between backs and forwards in Rugby Sevens. Points located to the right of the vertical line suggest that the incidence of injuries is higher among forwards compared to backs.

A total of seven studies investigated the incidence of injuries in Rugby Sevens. Forwards had a lower incidence of injuries overall when compared to backs (pooled IRR 0.78, CI 0.73–0.85, study *n* 7, *I* ^2^ = 6.6%) (Figure 4). Also, the incidence was lower for professional forwards (pooled IRR 0.79, CI 0.73–0.85, study *n* = 4, *I* ^2^ = 0.0%), but not for amateur players when compared to backs (Figures S29 and S30).

A total of six studies out of the seven studies in Rugby Sevens reported injuries in different anatomic locations, types of injuries, or causes of the injury. Forwards had a higher incidence of trunk and back injuries (pooled IRR 1.99, CI 1.42–2.78, study *n* = 6, *I* ^2^ = 73.1%), when compared to backs. Also, forwards had a lower incidence of arm and elbow injuries (pooled IRR 0.36, CI 0.17–0.76, study *n* = 4, *I* ^2^ = 0.0%), lower limb injuries (pooled IRR 0.74, CI 0.66–0.81, study *n* = 6, *I* ^2^ = 81.9%), knee injuries (pooled IRR 0.70, CI 0.57–0.85, study *n* = 4, *I* ^2^ = 81.5%), and foot and ankle injuries (pooled IRR 0.68, CI 0.56–0.82, study *n* = 46, *I* ^2^ = 0.0%), when compared to backs. In addition, forwards had a lower incidence of strain injuries (pooled IRR 0.59, CI 0.50–0.70, study *n* = 4, *I* ^2^ = 0.0%), injuries caused by tackling (pooled IRR 0.68, CI 0.61–0.76, study *n* = 4, *I* ^2^ = 89.9%), and injuries caused by running (pooled IRR 0.42, CI 0.33–0.52, study *n* = 4, *I* ^2^ = 89.9%), when compared to backs (Figures S31–S51 and Table 1).

### 3.6 Rugby League

**FIGURE 5:**
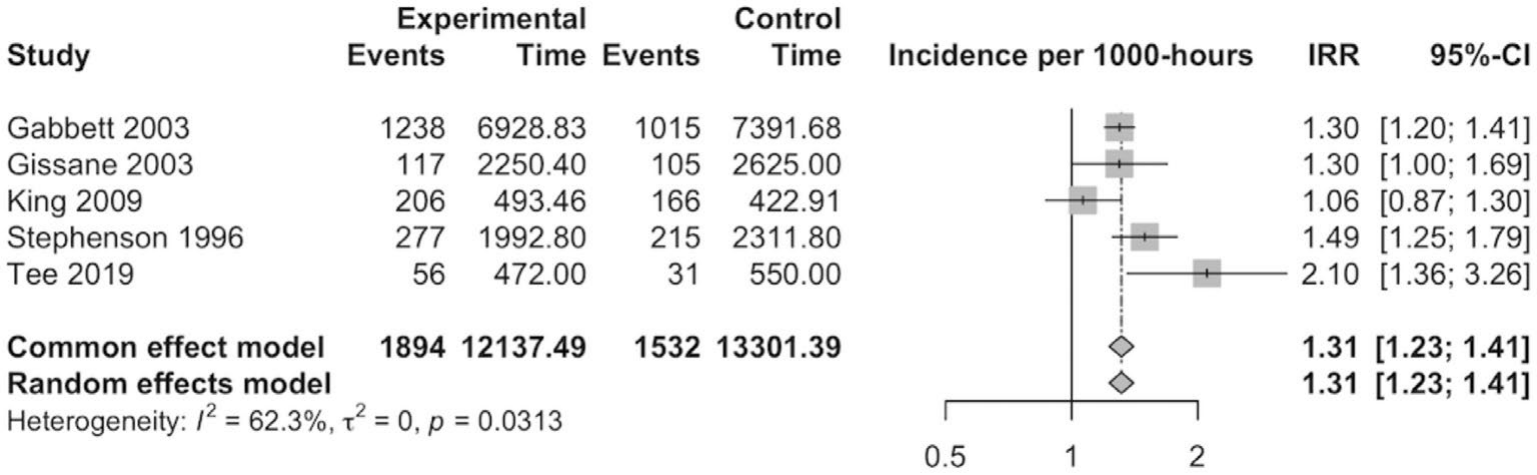
Incidence rate ratios (IRRs) with 95% confidence intervals (CI) for overall injuries between backs and forwards in Rugby League. Points located to the right of the vertical line suggest that the incidence of injuries is higher among forwards compared to backs.

A total of five studies investigated the incidence of injuries in Rugby League. Forwards had a higher incidence of injuries overall when compared to backs (pooled IRR 1.31, CI 1.23–1.41, study *n* 5, *I* ^2^ = 62.3%) (Figure 5). All studies reported only the overall number of injuries, and therefore additional analyses on the incidence of injuries by their location, nature, or cause were not performed.

### 3.7 Sensitivity Analysis

In the sensitivity analyses for medical attention injuries (Figure S52) and time‐loss injuries (Figure S53), forwards had a higher incidence of medical attention injuries (pooled IRR 1.22, CI 1.15–1.30, number of studies = 9, *I* ^2^ = 89.0%), but no evidence was found for time‐loss injuries (pooled IRR 0.99, CI 0.96–1.02, number of studies = 38, *I* ^2^ = 80.0%).

### 3.8 Publication Bias

Publication bias was evaluated using funnel plots. The plot suggests some mild asymmetry, which is most likely explained by known factors causing heterogeneity, including injury definitions, injury types, and rugby formats. However, as seen in the funnel plot, publication bias is very minimal (Figure S54).

## 4 Discussion

The main finding of this study was that a similar incidence of injuries was observed among both forwards and backs. However, there were major differences in different playing formats, in the anatomic locations, injury nature, and cause of the injury observed between playing positions. Forwards had a higher incidence of injuries in Rugby Union and Rugby League, whereas in Rugby Sevens, the incidence of injuries was markedly higher for backs. In addition, forwards had a higher incidence of trunk and back injuries in Rugby Union and Rugby Sevens, and backs had a higher incidence of injuries in the lower limb, especially in the knee, foot, and ankle in these formats. Backs also had a higher incidence of injuries caused by tackling or being tackled, running, or by collisions in both rugby formats.

Interestingly, on the contrary to the findings in Rugby Union and League, backs had a higher incidence of injuries in Rugby Sevens. While the exact reason for this remains unknown, this contrasting pattern could be explained by the unique physiological and tactical demands of the Rugby Sevens format. With fewer players on the field and significantly more open space, backs in Sevens are involved in more high‐speed running, cutting maneuvers, and one‐on‐one tackles than in other rugby formats. Backs are also required to cover more ground and engage in repeated high‐intensity efforts, contributing to fatigue‐related injuries in later stages of matches. Fuller et al. discussed that injury risk increases with match intensity and fatigue, especially for positions reliant on speed and agility [30]. Moreover, Rugby Sevens matches are typically played in tournament settings with multiple games per day, which may exacerbate cumulative fatigue and injury risk among backs.

In our study, forwards had markedly higher incidence of injuries of the back and torso in both formats (Rugby Union and Sevens) reporting anatomical locations of the injuries. While the exact reason remains unknown, these types of injuries could be linked to the physical demands of their role, where forwards often engage in high‐contact situations such as scrums, rucks, and mauls [10]. However, no evidence of difference was found in the injury incidence caused by rucks and mauls, but only four studies reported these, and these studies focused on all injuries caused by rucks and mauls, rather than only trunk and back injuries. Previous research has suggested that the high axial loading forces in scrums and collisions may contribute to the increased incidence of back injuries among forwards [68]. The greater height and mass of forwards might contribute to the increased risk of torso and back injuries, as their larger frames are subjected to more forceful impacts.

On the other hand, backs experienced a significantly higher incidence of muscle strain injuries and lower limb injuries, particularly knee and ankle injuries in Rugby Union and Rugby Sevens. This could be due to the nature of their position, where quick lateral movements, high‐speed sprints, and frequent tackles are more common [11, 12]. Backs also perform significantly more kicking than forwards, and this action is a recognized mechanism for common muscle strains, particularly in the hamstrings and quadriceps [69, 70]. Our dataset included 19 kicking‐related injuries, all occurring in backs, whereas no such injuries were recorded for attackers, precluding direct positional comparison in that context. Nonetheless, the presence of these injuries underscores the importance of addressing kicking‐related injury risks among backs. Also, the repeated stress on the muscles and joints of lower limbs, especially in the knees, might make backs more susceptible to such injuries. Additionally, the difference in playing styles where backs often rely on agility and explosive movements while forwards engage in more strength‐based, direct confrontations could explain these injury patterns [10]. This is supported by the finding that in both Rugby Union and Rugby Sevens, backs had a higher incidence of injuries caused by tackling, caused by running, or caused by collisions than forwards. However, based on our data it remains unknown whether the strain injuries occurred in the lower limb, as these were not stratified in the original studies. It is only evident that strain injuries, injuries in the lower limb, and certain injury mechanisms (sprints, tackles, kicking, and collisions) are more frequent among backs.

The results of this study suggest that a deeper understanding of these positional differences can inform targeted injury prevention programs. Deeper position‐specific literature on rugby injuries remains highly limited, especially in Rugby Sevens and Rugby League, as most studies only compare injury incidence between positions as a secondary analysis, without examining, for example, injury types or locations in relation to specific playing positions. In addition, the nature of the formats, such as the increased space and speed in Rugby Sevens compared to the greater physicality and set‐piece demands of Rugby Union, suggests that injury prevention strategies should be format‐specific and tailored not only to playing position but also to the distinct physical and tactical demands of each code. Therefore, more detailed research is urgently needed to examine injuries and their mechanisms across different rugby formats to support the development of effective, context‐specific interventions.

Recent studies have highlighted a high incidence of head injuries in rugby, often suggesting positional differences due to the distinct physical demands of forwards and backs [6]. Forwards engage in frequent collisions, scrums, and rucks, whereas backs experience high‐speed tackles and open‐field play [10, 11, 12, 13]. Interestingly, our study found no important differences in head injury, concussion, or injuries caused by scrums and rucks between the forwards and backs. One possible explanation is that while forwards endure more repetitive impacts, backs may sustain injuries from high‐velocity collisions, leading to a comparable overall risk [10, 11, 12, 13].

The pooled incidence of injuries was found to be high for both forwards and backs in our study, being approximately 23 per 1000 player‐hours for backs, and 25 per 1000 player‐hours for forwards. Despite the fact that pooled incidence in our study is higher than reported in most other sports, (e.g., ice hockey 5.9 per 1000 player‐hours, National Football League 19 per 1000 athlete‐exposures), previous literature has reported higher injury rates across all rugby formats [7, 8, 9, 71, 72, 73, 74]. Yeomans et al. investigating the incidence of injuries in amateur Rugby Union reported an incidence of 47 per 1000 player‐hours in 2018 [7]. Williams et al. reported incidence of professional Rugby Union injuries increasing as high as 91 per 1000 player‐hours in 2022 [8]. King et al. investigated the match and training injuries in Rugby League, which were reported to be 89 per 1000 player‐hours for match injuries, and 12 per 1000 player‐hours for training injuries in 2022 [9]. The exact reason for the slightly lower incidence of both playing positions in our study remains unknown. As stated above, the incidence of injuries varies a lot between different rugby formats, level of play, matches and training, and injury definitions [7, 8, 9]. Also in our review, the studies reporting higher incidences of rugby injuries were usually calculated using only match exposure, as training injuries are found to be much less common in all rugby formats [20, 26, 28, 29, 32, 40, 43, 44, 46, 54, 56, 58, 59, 65, 66]. In studies reporting incidences using exposure time including both match and training hours, the usually observed high number of training hours compared to match hours can be “covering” the actual high incidence in matches. For example, using only match‐hours, Gabbett et al. reported the injury incidence as high as 984 per 1000 match‐hours for forwards, and 702 per 1000 match‐hours for backs in semi‐professional Rugby Union [40]. Also, the definitions of injuries still varied between studies. King et al. reported a total incidence of 392 per 1000 player‐match‐hours for backs, and 417 player‐match‐hours for forwards, and the injury was defined as “any pain during the match irrespective of the need for match or training time loss or for first aid or medical attention” unlike in other studies [45]. These findings advocate, that in addition to position‐based stratification, research on rugby injury epidemiology require examination using more specific classification on the study populations, playing level, and comparable injury definitions (despite the raised concerns on inconsistencies already in 2007 by Fuller et al. [75]). Match and training injuries should be clearly analyzed separately and interpreted carefully. This topic is important, as especially high incidence of injuries in matches has been raising concerns in previous research during recent years across the rugby formats [67, 76].

The strengths of our study are its completeness and the number of included studies, as well as the use of player‐hours instead of athlete exposure. In addition, also match or training‐hours instead along with player‐hours were available for nearly all studies. Whereas our review may provide high quality estimates of the incidences and IRRs between forwards and backs for rugby injuries, with a truly high number of studies included and around million hours of exposure for both backs, and forwards, it also has a few limitations that should be addressed. The main limitation was that majority of studies were about Rugby Union, as the total number of studies reporting the player position based injury incidences in Rugby League or Sevens was truly low. In addition, none of the studies in Rugby League reported the number of injuries in different anatomic locations, nature of the injury, or the cause behind the injury, indicating that this topic should be further researched using position‐based stratification. Also, sensitivity analyses showed higher incidence of medical attention injuries, whereas no difference was found for time‐loss injuries This finding suggests that methodological or clinical factors may influence the observed differences. Medical attention injuries may be more susceptible to variation in reporting or clinical thresholds, potentially leading to systematic bias. For example, attackers may receive more frequent clinical assessments due to their game roles. These considerations underline the need to interpret medical attention injury data with caution, especially when comparing subgroups. To control the possible bias that the heterogeneity might have had on our results, we used the random‐effects models and presented our results in subgroups, which are relevant to the sport.

### 4.1 Perspective

In conclusion, the overall incidence of injuries was similar between forwards and backs. However, the location and nature of injuries varied by playing position. Forwards had a higher incidence of injuries in Rugby Union and Rugby League, whereas in Rugby Sevens, the incidence of injuries was markedly higher for backs. Forwards exhibited a significantly higher incidence of trunk and back injuries, whereas backs were more prone to lower limb injuries, particularly knee injuries and strains. In addition to a more standardized definition of injuries and more comprehensive subgroup analyses in future studies, different trends and patterns in injury occurrence regarding playing positions and all rugby formats should be further studied to develop more targeted injury prevention strategies in forwards and backs. In addition to position‐based stratification, research on rugby injury epidemiology requires examination using a more specific classification of study populations, playing levels, and comparable injury definitions, as methodological shortcomings have previously been highlighted as a concern in the literature [75].

## Author Contributions

Conceptualisation: M.V., J.L., I.K. Literature search: R.L. Abstract screening, full‐text eligibility assessment, data extraction, and critical appraisal: M.V., J.L., R.L., O.P., I.K. Data synthesis: M.V., J.L., R.L., O.P. Original draft of manuscript: M.V. Manuscript review and edit: M.V., J.L., R.L., O.P., I.K. All authors read and approved the final manuscript.

## Disclosure

Equity, Diversity and Inclusion: The research group includes two junior researchers. As the study was conducted as a systematic review and meta‐analysis, the data from the original studies were gathered as comprehensively as possible, and the results were reported based on the available data. The results for both genders were reported.

## Ethics Statement

The authors have nothing to report.

## Consent

The authors have nothing to report.

## Conflicts of Interest

The authors declare no conflicts of interest.

## Supporting information




Appendix S1.

Appendix S2.

Appendix S3.

Appendix S4.

## Data Availability

All data have been included in the article or included as Supporting Information.

## References

[1] World Rugby Passport , Laws of the Game (World Rugby, 2025), https://passport.world.rugby/laws‐of‐the‐game/.

[2] E. Daly , A. D. Blackett , A. J. Pearce , and L. Ryan , “Protect the Player, Protect the Game: Reflections From Ex‐Professional Rugby Union Players on Law Changes, Protective Equipment, and Duty of Care in the Professional Game,” Journal of Functional Morphology and Kinesiology 7 (2022): 91. 36278752 10.3390/jfmk7040091 PMC9624300

[3] D. G. Higham , D. B. Pyne , J. M. Anson , and A. Eddy , “Movement Patterns in Rugby Sevens: Effects of Tournament Level, Fatigue and Substitute Players,” Journal of Science and Medicine in Sport 15 (2012): 277–282. 22188846 10.1016/j.jsams.2011.11.256

[4] World Rugby , Overview (World Rugby, 2025), https://www.world.rugby/organisation/about‐us/overview.

[5] B. Cunniffe , W. Proctor , J. S. Baker , and B. Davies , “An Evaluation of the Physiological Demands of Elite Rugby Union Using Global Positioning System Tracking Software,” Journal of Strength and Conditioning Research 23 (2009): 1195–1203. 19528840 10.1519/JSC.0b013e3181a3928b

[6] S. W. West , L. Starling , S. Kemp , et al., “Trends in Match Injury Risk in Professional Male Rugby Union: A 16‐Season Review of 10 851 Match Injuries in the English Premiership (2002–2019): The Professional Rugby Injury Surveillance Project,” British Journal of Sports Medicine 55 (2021): 676–682. 33046453 10.1136/bjsports-2020-102529

[7] C. Yeomans , I. C. Kenny , R. Cahalan , et al., “The Incidence of Injury in Amateur Male Rugby Union: A Systematic Review and Meta‐Analysis,” Sports Medicine 48, no. 4 (2018): 837–848. 29299876 10.1007/s40279-017-0838-4 PMC5856893

[8] S. Williams , C. Robertson , L. Starling , et al., “Injuries in Elite Men's Rugby Union: An Updated (2012–2020) Meta‐Analysis of 11,620 Match and Training Injuries,” Sports Medicine 52 (2022): 1127–1140. 34854059 10.1007/s40279-021-01603-w PMC9023408

[9] D. A. King , T. N. Clark , P. A. Hume , and K. Hind , “Match and Training Injury Incidence in Rugby League: A Systematic Review, Pooled Analysis, and Update on Published Studies,” Sports Medicine and Health Science 4 (2022): 75–84. 35782281 10.1016/j.smhs.2022.03.002 PMC9219278

[10] World Rugby , Players and Positions (World Rugby, 2025), https://www.world.rugby/the‐game/beginners‐guide/positions.

[11] K. Till , S. Scantlebury , and B. Jones , “Anthropometric and Physical Qualities of Elite Male Youth Rugby League Players,” Sports Medicine 47 (2017): 2171–2186. 28578541 10.1007/s40279-017-0745-8 PMC5633637

[12] N. Dobbin , R. Hunwicks , J. Highton , and C. Twist , “A Reliable Testing Battery for Assessing Physical Qualities of Elite Academy Rugby League Players,” Journal of Strength and Conditioning Research 32 (2018): 3232–3238. 29140912 10.1519/JSC.0000000000002280

[13] C. W. Fuller , J. H. M. Brooks , R. J. Cancea , J. Hall , and S. P. T. Kemp , “Contact Events in Rugby Union and Their Propensity to Cause Injury,” British Journal of Sports Medicine 41 (2007): 862–867. 17513332 10.1136/bjsm.2007.037499 PMC2658974

[14] Z. Munn , S. Moola , D. Riitano , and K. Lisy , “The Development of a Critical Appraisal Tool for Use in Systematic Reviews Addressing Questions of Prevalence,” International Journal of Health Policy and Management 3 (2014): 123–128. 25197676 10.15171/ijhpm.2014.71 PMC4154549

[15] J. P. T. Higgins , J. Thomas , J. Chandler , et al., Cochrane Handbook for Systematic Reviews of Interventions, 2nd ed. (Wiley, 2019).

[16] S. Balduzzi , G. Rücker , and G. Schwarzer , “How to Perform a Meta‐Analysis With R: A Practical Tutorial,” Evidence‐Based Mental Health 22 (2019): 153–160. 31563865 10.1136/ebmental-2019-300117 PMC10231495

[17] G. Schwarzer , “meta: An R Package for Meta‐Analysis,” Version [4.21.2]. R Package, 2025, https://cran.r‐project.org/package=meta.

[18] S. J. Bailey , R. Martindale , L. Engebretsen , J. P. Robson , and D. Palmer , “Epidemiology of International Match Injuries in Scottish Rugby: A Prospective Cohort Study,” International Journal of Sports Medicine 44 (2023): 805–812. 37279793 10.1055/a-2038-3452

[19] C. L. Bitchell , P. Mathema , and I. S. Moore , “Four‐Year Match Injury Surveillance in Male Welsh Professional Rugby Union Teams,” Physical Therapy in Sport 42 (2020): 26–32. 31869752 10.1016/j.ptsp.2019.12.001

[20] J. H. M. Brooks , C. W. Fuller , S. P. T. Kemp , and D. B. Reddin , “Epidemiology of Injuries in English Professional Rugby Union: Part 2 Training Injuries,” British Journal of Sports Medicine 39 (2005): 767–775. 16183775 10.1136/bjsm.2005.018408 PMC1725038

[21] J. H. M. Brooks , C. W. Fuller , S. P. T. Kemp , and D. B. Reddin , “A Prospective Study of Injuries and Training Amongst the England 2003 Rugby World Cup Squad,” British Journal of Sports Medicine 39 (2005): 288–293. 15849293 10.1136/bjsm.2004.013391 PMC1725216

[22] J. H. M. Brooks and S. P. T. Kemp , “Injury‐Prevention Priorities According to Playing Position in Professional Rugby Union Players,” British Journal of Sports Medicine 45 (2011): 765–775. 20484316 10.1136/bjsm.2009.066985

[23] J. Chéradame , R. Loursac , J. Piscione , C. Carling , P. Decq , and H. Jacqmin‐Gadda , “Impact of Weekly Training‐Load Structure and Content on the Risk of Injury in Professional Rugby Union Match‐Play,” Journal of Strength and Conditioning Research 38 (2024): 1613–1619. 39074175 10.1519/JSC.0000000000004852

[24] M. Cosgrave and S. Williams , “The Epidemiology of Concussion in Professional Rugby Union in Ireland,” Physical Therapy in Sport 35 (2019): 99–105. 30513491 10.1016/j.ptsp.2018.11.010

[25] A. M. Cruz‐Ferreira , E. M. Cruz‐Ferreira , P. B. Ribeiro , L. M. Santiago , and L. Taborda‐Barata , “Epidemiology of Time‐Loss Injuries in Senior and Under‐18 Portuguese Male Rugby Players,” Journal of Human Kinetics 62 (2018): 73–80. 29922379 10.1515/hukin-2017-0159 PMC6006535

[26] S. L. Evans , O. E. Davis , E. S. Jones , J. Hardy , and J. A. Owen , “Match and Training Injury Risk in Semi‐Professional Rugby Union: A Four‐Year Study,” Journal of Science and Medicine in Sport 25 (2022): 379–384. 35184953 10.1016/j.jsams.2022.01.003

[27] T. Farley , E. Barry , K. Bester , et al., “Poor Cervical Proprioception as a Risk Factor for Concussion in Professional Male Rugby Union Players,” Physical Therapy in Sport 55 (2022): 211–217. 35526515 10.1016/j.ptsp.2022.03.010

[28] C. W. Fuller , J. H. M. Brooks , and S. P. T. Kemp , “Spinal Injuries in Professional Rugby Union: A Prospective Cohort Study,” Clinical Journal of Sport Medicine 17 (2007): 10–16. 17304000 10.1097/JSM.0b013e31802e9c28

[29] C. W. Fuller , F. Laborde , R. J. Leather , and M. G. Molloy , “International Rugby Board Rugby World Cup 2007 Injury Surveillance Study,” British Journal of Sports Medicine 42 (2008): 452–459. 18539659 10.1136/bjsm.2008.047035

[30] C. W. Fuller , A. Taylor , and M. G. Molloy , “Epidemiological Study of Injuries in International Rugby Sevens,” Clinical Journal of Sport Medicine 20 (2010): 179–184. 20445357 10.1097/JSM.0b013e3181df1eea

[31] C. W. Fuller , M. G. Molloy , and M. Marsalli , “Epidemiological Study of Injuries in Men's International Under‐20 Rugby Union Tournaments,” Clinical Journal of Sport Medicine: Official Journal of the Canadian Academy of Sport Medicine 21 (2011): 356–358. 21617525 10.1097/JSM.0b013e31821f5085

[32] C. W. Fuller , K. Sheerin , and S. Targett , “Rugby World Cup 2011: International Rugby Board Injury Surveillance Study,” British Journal of Sports Medicine 47 (2013): 1184–1191. 22685124 10.1136/bjsports-2012-091155

[33] C. W. Fuller , A. E. Taylor , and M. Raftery , “Should Player Fatigue Be the Focus of Injury Prevention Strategies for International Rugby Sevens Tournaments?,” British Journal of Sports Medicine 50 (2016): 682–687. 27190230 10.1136/bjsports-2016-096043

[34] C. W. Fuller , A. Taylor , S. P. T. Kemp , and M. Raftery , “Rugby World Cup 2015: World Rugby Injury Surveillance Study,” British Journal of Sports Medicine 51 (2017): 51–57. 27461882 10.1136/bjsports-2016-096275

[35] C. W. Fuller , “A Kinetic Model Describing Injury‐Burden in Team Sports,” Sports Medicine 47 (2017): 2641–2651. 28573403 10.1007/s40279-017-0746-7

[36] C. W. Fuller , A. Taylor , and M. Raftery , “Eight‐Season Epidemiological Study of Injuries in Men's International Under‐20 Rugby Tournaments,” Journal of Sports Sciences 36 (2018): 1776–1783. 29252097 10.1080/02640414.2017.1418193

[37] C. W. Fuller , A. Taylor , M. Douglas , and M. Raftery , “Rugby World Cup 2019 Injury Surveillance Study,” South African Journal of Sports Medicine 32 (2020): v32i1a8062. 10.17159/2078-516X/2020/v32i1a8062 PMC9924538 36818969

[38] C. W. Fuller and A. Taylor , “Ten‐Season Epidemiological Study of Match Injuries in Men's International Rugby Sevens,” Journal of Sports Sciences 38 (2020): 1595–1604. 32286146 10.1080/02640414.2020.1752059

[39] C. W. Fuller and A. Taylor , “Eight‐Season Epidemiological Study of Match Injuries in Women's International Rugby Sevens,” Journal of Sports Sciences 39 (2021): 865–874. 33225825 10.1080/02640414.2020.1850616

[40] T. J. Gabbett , “Incidence of Injury in Semi‐Professional Rugby League Players,” British Journal of Sports Medicine 37 (2003): 36–43, discussion 43–44. 12547741 10.1136/bjsm.37.1.36 PMC1724588

[41] C. Gissane , D. Jennings , K. Kerr , and J. White , “Injury Rates in Rugby League Football: Impact of Change in Playing Season,” American Journal of Sports Medicine 31 (2003): 954–958. 14623663 10.1177/03635465030310063501

[42] M. V. Hancock , C. Barden , S. P. Roberts , C. D. McKay , and K. A. Stokes , “Match Injuries in English Schoolboy Rugby Union,” BMJ Open Sport & Exercise Medicine 10 (2024): e001740. 10.1136/bmjsem-2023-001740 PMC11148659 38268528

[43] J. Headey , J. H. M. Brooks , and S. P. T. Kemp , “The Epidemiology of Shoulder Injuries in English Professional Rugby Union,” American Journal of Sports Medicine 35 (2007): 1537–1543. 17452514 10.1177/0363546507300691

[44] S. P. T. Kemp , Z. Hudson , J. H. M. Brooks , and C. W. Fuller , “The Epidemiology of Head Injuries in English Professional Rugby Union,” Clinical Journal of Sport Medicine 18 (2008): 227–234. 18469563 10.1097/JSM.0b013e31816a1c9a

[45] D. A. King and C. Gissane , “Injuries in Amateur Rugby League Matches in New Zealand: A Comparison Between a Division 1 and a Division 2 Premier Grade Team,” Clinical Journal of Sport Medicine 19 (2009): 277–281. 19638820 10.1097/JSM.0b013e3181a7c6b0

[46] D. King , P. A. Hume , T. Clark , A. Foskett , and M. J. Barnes , “Training Injury Incidence in an Amateur Women's Rugby Union Team in New Zealand Over Two Consecutive Seasons,” Journal of Science and Medicine in Sport 24 (2021): 544–548. 33243595 10.1016/j.jsams.2020.11.005

[47] T. M. Leahy , I. C. Kenny , M. J. Campbell , et al., “Injury Trends for School Rugby Union in Ireland: The Need for Position‐Specific Injury‐Prevention Programs,” Sports Health 15 (2023): 131–141. 35354389 10.1177/19417381221078531 PMC9808841

[48] N. P. S. Lopes , A. M. Cruz‐Ferreira , D. T. Lima , M. A. Silva , and L. M. Santiago , “Athlete Health Implications of Match Injuries in Portuguese Rugby Union,” International Journal of Environmental Research and Public Health 21 (2024): 1191. 39338074 10.3390/ijerph21091191 PMC11430984

[49] V. Lopez , G. J. Galano , C. M. Black , et al., “Profile of an American Amateur Rugby Union Sevens Series,” American Journal of Sports Medicine 40 (2012): 179–184. 22102102 10.1177/0363546511427124

[50] V. Lopez , R. Ma , M. G. Weinstein , et al., “United States Under‐19 Rugby‐7s: Incidence and Nature of Match Injuries During a 5‐Year Epidemiological Study,” Sports Medicine 6 (2020): 41. 10.1186/s40798-020-00261-y PMC7452962 32852666

[51] R. Ma , V. Lopez , M. G. Weinstein , et al., “Injury Profile of American Women's Rugby‐7s,” Medicine and Science in Sports and Exercise 48 (2016): 1957–1966. 27232243 10.1249/MSS.0000000000000997

[52] S. Mc Fie , J. Brown , S. Hendricks , et al., “Incidence and Factors Associated With Concussion Injuries at the 2011 to 2014 South African Rugby Union Youth Week Tournaments,” Clinical Journal of Sport Medicine 26 (2016): 398–404. 27604072 10.1097/JSM.0000000000000276

[53] N. Muma , H. Saidi , and J. W. Githaiga , “Surveillance of Injuries Among Kenya Rugby Union (KRU) Players: Season 2010,” Annals of African Surgery 9 (2012): 88–92.

[54] R. Murias‐Lozano , L. Mendía , F. J. S. Sebastián‐Obregón , et al., “The Epidemiology of Injuries in Spanish Rugby Union División de Honor,” International Journal of Environmental Research and Public Health 19 (2022): 3882. 35409565 10.3390/ijerph19073882 PMC8997440

[55] S. Murray‐Smith , S. Williams , M. Whalan , G. E. Peoples , and J. A. Sampson , “The Incidence and Burden of Injury in Male Adolescent Community Rugby Union in Australia,” Science and Medicine in Football 7 (2023): 315–322. 36134642 10.1080/24733938.2022.2123556

[56] C. J. Pearce , J. H. M. Brooks , S. P. T. Kemp , and J. D. F. Calder , “The Epidemiology of Foot Injuries in Professional Rugby Union Players,” Foot and Ankle Surgery 17 (2011): 113–118. 21783068 10.1016/j.fas.2010.02.004

[57] D. M. Schick , M. G. Molloy , and J. P. Wiley , “Injuries During the 2006 Women's Rugby World Cup,” British Journal of Sports Medicine 42 (2008): 447–451. 18424486 10.1136/bjsm.2008.046672

[58] M. P. Schwellnus , A. Thomson , W. Derman , et al., “More Than 50% of Players Sustained a Time‐Loss Injury (>1 Day of Lost Training or Playing Time) During the 2012 Super Rugby Union Tournament: A Prospective Cohort Study of 17,340 Player‐Hours,” British Journal of Sports Medicine 48 (2014): 1306–1315. 24982503 10.1136/bjsports-2014-093745

[59] M. P. Schwellnus , E. Jordaan , C. Janse van Rensburg , et al., “Match Injury Incidence During the Super Rugby Tournament Is High: A Prospective Cohort Study Over Five Seasons Involving 93 641 Player‐Hours,” British Journal of Sports Medicine 53 (2019): 620–627. 29959135 10.1136/bjsports-2018-099105

[60] L. T. Starling , N. Gabb , S. Williams , S. Kemp , and K. A. Stokes , “Longitudinal Study of Six Seasons of Match Injuries in Elite Female Rugby Union,” British Journal of Sports Medicine 57 (2023): 212–217. 36428090 10.1136/bjsports-2022-105831

[61] S. Stephenson , C. Gissane , and D. Jennings , “Injury in Rugby League: A Four Year Prospective Survey,” British Journal of Sports Medicine 30 (1996): 331–334. 9015597 10.1136/bjsm.30.4.331 PMC1332420

[62] M. S. Swain , H. P. Pollard , and R. Bonello , “Incidence, Severity, Aetiology and Type of Neck Injury in Men's Amateur Rugby Union: A Prospective Cohort Study,” Chiropractic & Osteopathy 18 (2010): 18. 20594296 10.1186/1746-1340-18-18 PMC2907385

[63] M. S. Swain , R. P. Lystad , N. Henschke , C. G. Maher , and S. J. Kamper , “Match Injuries in Amateur Rugby Union: A Prospective Cohort Study ‐ FICS Biennial Symposium Second Prize Research Award,” Chiropractic & Manual Therapies 24 (2016): 17. 27252828 10.1186/s12998-016-0098-7 PMC4888508

[64] J. C. Tee , K. Till , and B. Jones , “Incidence and Characteristics of Injury in Under‐19 Academy Level Rugby League Match Play: A Single Season Prospective Cohort Study,” Journal of Sports Sciences 37 (2019): 1181–1188. 30430907 10.1080/02640414.2018.1547100

[65] E. Tondelli , S. Zabaloy , T. M. Comyns , and I. C. Kenny , “Effect of COVID‐19 Lockdown on Injury Incidence and Burden in Amateur Rugby Union,” Physical Therapy in Sport 59 (2023): 85–91. 36525741 10.1016/j.ptsp.2022.12.005 PMC9737509

[66] T. Whitehouse , R. Orr , E. Fitzgerald , S. Harries , and C. P. McLellan , “The Epidemiology of Injuries in Australian Professional Rugby Union 2014 Super Rugby Competition,” Orthopaedic Journal of Sports Medicine 4 (2016): 2325967116634075. 27069947 10.1177/2325967116634075 PMC4811007

[67] A. M. Cruz‐Ferreira , A. Montocchio , E. Usova‐Akula , P. Tuccelli , and F. Marty , “2021/22 Rugby Europe Injury Surveillance Report: SuperCup, Under‐20, and Under‐18 Championship,” International Journal of Environmental Research and Public Health 20 (2023): 1800. 36767165 10.3390/ijerph20031800 PMC9914350

[68] E. Preatoni , K. A. Stokes , M. E. England , and G. Trewartha , “The Influence of Playing Level on the Biomechanical Demands Experienced by Rugby Union Forwards During Machine Scrummaging,” Scandinavian Journal of Medicine & Science in Sports 23 (2013): e178–e184. 23362799 10.1111/sms.12048

[69] F. Kerin , G. Farrell , P. Tierney , U. McCarthy Persson , G. De Vito , and E. Delahunt , “Its Not All About Sprinting: Mechanisms of Acute Hamstring Strain Injuries in Professional Male Rugby Union—A Systematic Visual Video Analysis,” British Journal of Sports Medicine 56 (2022): 608–615. 35045971 10.1136/bjsports-2021-104171

[70] S. L. Lazarczuk , T. Love , M. J. Cross , et al., “The Epidemiology of Kicking Injuries in Professional Rugby Union: A 15‐Season Prospective Study,” Scandinavian Journal of Medicine & Science in Sports 30 (2020): 1739–1747. 32492220 10.1111/sms.13737

[71] I. Kuitunen , V. Immonen , O. Pakarinen , V. M. Mattila , and V. T. Ponkilainen , “Incidence of Football Injuries Sustained on Artificial Turf Compared to Grass and Other Playing Surfaces: A Systematic Review and Meta‐Analysis,” EClinicalMedicine 59 (2023): 101956. 37125402 10.1016/j.eclinm.2023.101956 PMC10139885

[72] L. C. Ladehoff , D. Kuruvilla , E. Coughlin , R. Mhaskar , and D. T. Remaley , “Epidemiology of American Football‐Related Fractures in the United States 2002–2021,” Orthopaedic Journal of Sports Medicine 12, no. 9 (2024): 23259671241259481. 39253289 10.1177/23259671241259481 PMC11382224

[73] G. Ornon , J. L. Ziltener , D. Fritschy , and J. Menetrey , “Epidemiology of Injuries in Professional Ice Hockey: A Prospective Study Over Seven Years,” Journal of Experimental Orthopaedics 7 (2020): 87. 33159261 10.1186/s40634-020-00300-3 PMC7647969

[74] H. S. Angileri , D. E. McLoughlin , M. M. Owen , J. M. May , M. A. Terry , and V. K. Tjong , “Association of Injury Rates Among Players in the National Football League With Playoff Qualification, Travel Distance, Game Timing, and the Addition of Another Game: Data From the 2017 to 2022 Seasons,” Orthopaedic Journal of Sports Medicine 11 (2023): 23259671231177633. 37547079 10.1177/23259671231177633 PMC10399261

[75] C. W. Fuller , M. G. Molloy , C. Bagate , et al., “Consensus Statement on Injury Definitions and Data Collection Procedures for Studies of Injuries in Rugby Union,” British Journal of Sports Medicine 41 (2007): 328–331. 17452684 10.1136/bjsm.2006.033282 PMC2659070

[76] G. J. Farah , B. C. Mitchell , M. R. Schmitz , J. D. Bomar , and E. W. Edmonds , “Injury Patterns in Rugby Union—America's Fastest Growing Sport,” Journal of the Pediatric Orthopaedic Society of North America 4 (2022): 406.

