# Supplementary material for: Differences in Injury Incidence Between Player Positions Across All Rugby Formats—A Systematic Review and Meta‐Analysis
Source: Scand J Med Sci Sports. 2025 Jul 7;35(7):e70102. doi: 10.1111/sms.70102 (PMC12233056; doi:10.1111/sms.70102)
Supplement: Supplementary file 2 — Appendix S2. [file SMS-35-e70102-s002.pdf]

Codes used for calculating the incidence rate ratios (IRRs)

~~~~
```{r}
library(,dyverse)
library(meta)
library(ggpubr)
library(readxl)
library(gt)
library(patchwork)
```
```{r}
Data_matrix <- read_excel("/Users/qsmava/Desktop/Rugbydata.xlsx",
sheet = "IRR") %>%
mutate(par,cipants_H = as.numeric(par,cipants_H)) %>%
mutate(injuries_overall_H = as.numeric(injuries_overall_H)) %>%
mutate(exposure_overall_H = as.numeric(exposure_overall_H)) %>%
mutate(injuries_training_H = as.numeric(injuries_training_H)) %>%
mutate(exposure_training_H = as.numeric(exposure_training_H)) %>%
mutate(injuries_match_H = as.numeric(injuries_match_H)) %>%
mutate(exposure_match_H = as.numeric(exposure_match_H)) %>%
mutate(par,cipants_B = as.numeric(par,cipants_B)) %>%
mutate(injuries_overall_B = as.numeric(injuries_overall_B)) %>%
mutate(exposure_overall_B = as.numeric(exposure_overall_B)) %>%
mutate(injuries_training_B = as.numeric(injuries_training_B)) %>%
mutate(exposure_training_B = as.numeric(exposure_training_B)) %>%
mutate(injuries_match_B = as.numeric(injuries_match_B)) %>%
mutate(exposure_match_B = as.numeric(exposure_match_B)) %>%
drop_na(study) %>%
filter(format == "League") %>% (Choose format)
#drop_na(collision_H) (Choose injury type)
meta_analysis <- metainc(event.e = injuries_overall_H, (Injury type for forwards)
,me.e = exposure_overall_H, (Exposure type for forwards)
event.c = injuries_overall_B, (Injury type for backs)
,me.c = exposure_overall_B, (Exposure type for backs)
studlab = study,
method = "GLMM",
sm = "IRR",
data = Data_matrix)
summary(meta_analysis)
Fig <- forest(meta_analysis,
pooled.,mes = T,
pooled.events = T,
pooled.totals = T,
smlab = "Incidence per 1000-hours")
```
~~~~
