# Supplementary material for: Differences in Injury Incidence Between Player Positions Across All Rugby Formats—A Systematic Review and Meta‐Analysis
Source: Scand J Med Sci Sports. 2025 Jul 7;35(7):e70102. doi: 10.1111/sms.70102 (PMC12233056; doi:10.1111/sms.70102)
Supplement: Supplementary file 3 — Appendix S3. [file SMS-35-e70102-s004.docx]

**SUPPLEMENTARY MATERIALS**

Contents

[Supplementary figure 1: The pooled injury incidence (vertical dashed line) with 95% confidence intervals (CI) (Horizontal line around the absolute incidence for each study) of rugby injuries for forwards. 6](#_Toc198120388)

[Supplementary figure 2: The pooled injury incidence (vertical dashed line) with 95% confidence intervals (CI) (Horizontal line around the absolute incidence for each study) of rugby injuries for backs. 7](#_Toc198120389)

[Supplementary figure 3: Incidence rate ratios (IRRs) with 95% confidence intervals (CI) for training injuries between backs and forwards in rugby union. Points located to the right of the vertical line suggest that the incidence of injuries is higher among forwards compared to backs. 8](#_Toc198120390)

[Supplementary figure 4: Incidence rate ratios (IRRs) with 95% confidence intervals (CI) for match injuries between backs and forwards in rugby union. Points located to the right of the vertical line suggest that the incidence of injuries is higher among forwards compared to backs. 9](#_Toc198120391)

[Supplementary figure 5: Incidence rate ratios (IRRs) with 95% confidence intervals (CI) for overall injuries between professional backs and forwards in rugby union. Points located to the right of the vertical line suggest that the incidence of injuries is higher among forwards compared to backs. 10](#_Toc198120392)

[Supplementary figure 6: Incidence rate ratios (IRRs) with 95% confidence intervals (CI) for overall injuries between amateur backs and forwards in rugby union. Points located to the right of the vertical line suggest that the incidence of injuries is higher among forwards compared to backs. 11](#_Toc198120393)

[Supplementary figure 7: Incidence rate ratios (IRRs) with 95% confidence intervals (CI) for head injuries between backs and forwards in rugby union. Points located to the right of the vertical line suggest that the incidence of injuries is higher among forwards compared to backs. 12](#_Toc198120394)

[Supplementary figure 8: Incidence rate ratios (IRRs) with 95% confidence intervals (CI) for upper limb injuries between backs and forwards in rugby union. Points located to the right of the vertical line suggest that the incidence of injuries is higher among forwards compared to backs. 13](#_Toc198120395)

[Supplementary figure 9: Incidence rate ratios (IRRs) with 95% confidence intervals (CI) for shoulder injuries between backs and forwards in rugby union. Points located to the right of the vertical line suggest that the incidence of injuries is higher among forwards compared to backs. 14](#_Toc198120396)

[Supplementary figure 10: Incidence rate ratios (IRRs) with 95% confidence intervals (CI) for arm and elbow injuries between backs and forwards in rugby union. Points located to the right of the vertical line suggest that the incidence of injuries is higher among forwards compared to backs. 15](#_Toc198120397)

[Supplementary figure 11: Incidence rate ratios (IRRs) with 95% confidence intervals (CI) for forearm and wrist injuries between backs and forwards in rugby union. Points located to the right of the vertical line suggest that the incidence of injuries is higher among forwards compared to backs. 16](#_Toc198120398)

[Supplementary figure 12: Incidence rate ratios (IRRs) with 95% confidence intervals (CI) for trunk and back injuries between backs and forwards in rugby union 17](#_Toc198120399)

[Supplementary figure 13: Incidence rate ratios (IRRs) with 95% confidence intervals (CI) for lower limb injuries between backs and forwards in rugby union. Points located to the right of the vertical line suggest that the incidence of injuries is higher among forwards compared to backs. 18](#_Toc198120400)

[Supplementary figure 14: Incidence rate ratios (IRRs) with 95% confidence intervals (CI) for hip and groin injuries between backs and forwards in rugby union. Points located to the right of the vertical line suggest that the incidence of injuries is higher among forwards compared to backs. 19](#_Toc198120401)

[Supplementary figure 15: Incidence rate ratios (IRRs) with 95% confidence intervals (CI) for thigh injuries between backs and forwards in rugby union. Points located to the right of the vertical line suggest that the incidence of injuries is higher among forwards compared to backs. 20](#_Toc198120402)

[Supplementary figure 16: Incidence rate ratios (IRRs) with 95% confidence intervals (CI) for calf injuries between backs and forwards in rugby union. Points located to the right of the vertical line suggest that the incidence of injuries is higher among forwards compared to backs. 21](#_Toc198120403)

[Supplementary figure 17: Incidence rate ratios (IRRs) with 95% confidence intervals (CI) for knee injuries between backs and forwards in rugby union. Points located to the right of the vertical line suggest that the incidence of injuries is higher among forwards compared to backs. 22](#_Toc198120404)

[Supplementary figure 18: Incidence rate ratios (IRRs) with 95% confidence intervals (CI) for foot and ankle injuries between backs and forwards in rugby union. Points located to the right of the vertical line suggest that the incidence of injuries is higher among forwards compared to backs. 23](#_Toc198120405)

[Supplementary figure 19: Incidence rate ratios (IRRs) with 95% confidence intervals (CI) for concussions between backs and forwards in rugby union. Points located to the right of the vertical line suggest that the incidence of injuries is higher among forwards compared to backs. 24](#_Toc198120406)

[Supplementary figure 20: Incidence rate ratios (IRRs) with 95% confidence intervals (CI) for sprain injuries between backs and forwards in rugby union. Points located to the right of the vertical line suggest that the incidence of injuries is higher among forwards compared to backs. 25](#_Toc198120407)

[Supplementary figure 21: Incidence rate ratios (IRRs) with 95% confidence intervals (CI) for strain injuries between backs and forwards in rugby union. Points located to the right of the vertical line suggest that the incidence of injuries is higher among forwards compared to backs. 26](#_Toc198120408)

[Supplementary figure 22: Incidence rate ratios (IRRs) with 95% confidence intervals (CI) for skin laceration injuries between backs and forwards in rugby union. Points located to the right of the vertical line suggest that the incidence of injuries is higher among forwards compared to backs. 27](#_Toc198120409)

[Supplementary figure 23: Incidence rate ratios (IRRs) with 95% confidence intervals (CI) for nerve injuries (excluding concussions) between backs and forwards in rugby union. Points located to the right of the vertical line suggest that the incidence of injuries is higher among forwards compared to backs. 28](#_Toc198120410)

[Supplementary figure 24: Incidence rate ratios (IRRs) with 95% confidence intervals (CI) for bone injuries between backs and forwards in rugby union. Points located to the right of the vertical line suggest that the incidence of injuries is higher among forwards compared to backs. 29](#_Toc198120411)

[Supplementary figure 25: Incidence rate ratios (IRRs) with 95% confidence intervals (CI) for tackle injuries between backs and forwards in rugby union. Points located to the right of the vertical line suggest that the incidence of injuries is higher among forwards compared to backs. 30](#_Toc198120412)

[Supplementary figure 26: Incidence rate ratios (IRRs) with 95% confidence intervals (CI) for ruck and maul injuries between backs and forwards in rugby union. Points located to the right of the vertical line suggest that the incidence of injuries is higher among forwards compared to backs. 31](#_Toc198120413)

[Supplementary figure 27: Incidence rate ratios (IRRs) with 95% confidence intervals (CI) for running injuries between backs and forwards in rugby union. Points located to the right of the vertical line suggest that the incidence of injuries is higher among forwards compared to backs. 32](#_Toc198120414)

[Supplementary figure 28: Incidence rate ratios (IRRs) with 95% confidence intervals (CI) for collision injuries between backs and forwards in rugby union. Points located to the right of the vertical line suggest that the incidence of injuries is higher among forwards compared to backs. 33](#_Toc198120415)

[Supplementary figure 29: Incidence rate ratios (IRRs) with 95% confidence intervals (CI) for overall injuries between professional backs and forwards in rugby seven. Points located to the right of the vertical line suggest that the incidence of injuries is higher among forwards compared to backs. 34](#_Toc198120416)

[Supplementary figure 30: Incidence rate ratios (IRRs) with 95% confidence intervals (CI) for overall injuries between amateur backs and forwards in rugby seven. Points located to the right of the vertical line suggest that the incidence of injuries is higher among forwards compared to backs. 35](#_Toc198120417)

[Supplementary figure 31: Incidence rate ratios (IRRs) with 95% confidence intervals (CI) for head injuries between backs and forwards in rugby seven. Points located to the right of the vertical line suggest that the incidence of injuries is higher among forwards compared to backs. 36](#_Toc198120418)

[Supplementary figure 32: Incidence rate ratios (IRRs) with 95% confidence intervals (CI) for upper limb injuries between backs and forwards in rugby seven. Points located to the right of the vertical line suggest that the incidence of injuries is higher among forwards compared to backs. 37](#_Toc198120419)

[Supplementary figure 33: Incidence rate ratios (IRRs) with 95% confidence intervals (CI) for shoulder injuries between backs and forwards in rugby seven. Points located to the right of the vertical line suggest that the incidence of injuries is higher among forwards compared to backs. 38](#_Toc198120420)

[Supplementary figure 34: Incidence rate ratios (IRRs) with 95% confidence intervals (CI) for arm and elbow injuries between backs and forwards in rugby seven. Points located to the right of the vertical line suggest that the incidence of injuries is higher among forwards compared to backs. 39](#_Toc198120421)

[Supplementary figure 35: Incidence rate ratios (IRRs) with 95% confidence intervals (CI) for forearm and wrist injuries between backs and forwards in rugby seven. Points located to the right of the vertical line suggest that the incidence of injuries is higher among forwards compared to backs. 40](#_Toc198120422)

[Supplementary figure 36: Incidence rate ratios (IRRs) with 95% confidence intervals (CI) for trunk and back injuries between backs and forwards in rugby seven. Points located to the right of the vertical line suggest that the incidence of injuries is higher among forwards compared to backs. 41](#_Toc198120423)

[Supplementary figure 37: Incidence rate ratios (IRRs) with 95% confidence intervals (CI) for lower limb injuries between backs and forwards in rugby union. Points located to the right of the vertical line suggest that the incidence of injuries is higher among forwards compared to backs. 42](#_Toc198120424)

[Supplementary figure 38: Incidence rate ratios (IRRs) with 95% confidence intervals (CI) for hip and groin injuries between backs and forwards in rugby union. Points located to the right of the vertical line suggest that the incidence of injuries is higher among forwards compared to backs. 43](#_Toc198120425)

[Supplementary figure 39: Incidence rate ratios (IRRs) with 95% confidence intervals (CI) for thigh injuries between backs and forwards in rugby union. Points located to the right of the vertical line suggest that the incidence of injuries is higher among forwards compared to backs. 44](#_Toc198120426)

[Supplementary figure 40: Incidence rate ratios (IRRs) with 95% confidence intervals (CI) for knee injuries between backs and forwards in rugby union. Points located to the right of the vertical line suggest that the incidence of injuries is higher among forwards compared to backs. 45](#_Toc198120427)

[Supplementary figure 41: Incidence rate ratios (IRRs) with 95% confidence intervals (CI) for foot and ankle injuries between backs and forwards in rugby union. Points located to the right of the vertical line suggest that the incidence of injuries is higher among forwards compared to backs. 46](#_Toc198120428)

[Supplementary figure 42: Incidence rate ratios (IRRs) with 95% confidence intervals (CI) for concussions between backs and forwards in rugby union. Points located to the right of the vertical line suggest that the incidence of injuries is higher among forwards compared to backs. 47](#_Toc198120429)

[Supplementary figure 43: Incidence rate ratios (IRRs) with 95% confidence intervals (CI) for sprain injuries between backs and forwards in rugby union. Points located to the right of the vertical line suggest that the incidence of injuries is higher among forwards compared to backs. 48](#_Toc198120430)

[Supplementary figure 44: Incidence rate ratios (IRRs) with 95% confidence intervals (CI) for strain injuries between backs and forwards in rugby union. Points located to the right of the vertical line suggest that the incidence of injuries is higher among forwards compared to backs. 49](#_Toc198120431)

[Supplementary figure 45: Incidence rate ratios (IRRs) with 95% confidence intervals (CI) for skin laceration injuries between backs and forwards in rugby union. Points located to the right of the vertical line suggest that the incidence of injuries is higher among forwards compared to backs. 50](#_Toc198120432)

[Supplementary figure 46: Incidence rate ratios (IRRs) with 95% confidence intervals (CI) for nerve injuries (excluding concussions) between backs and forwards in rugby union. Points located to the right of the vertical line suggest that the incidence of injuries is higher among forwards compared to backs. 51](#_Toc198120433)

[Supplementary figure 47: Incidence rate ratios (IRRs) with 95% confidence intervals (CI) for bone injuries between backs and forwards in rugby union. Points located to the right of the vertical line suggest that the incidence of injuries is higher among forwards compared to backs. 52](#_Toc198120434)

[Supplementary figure 48: Incidence rate ratios (IRRs) with 95% confidence intervals (CI) for tackle injuries between backs and forwards in rugby union. Points located to the right of the vertical line suggest that the incidence of injuries is higher among forwards compared to backs. 53](#_Toc198120435)

[Supplementary figure 49: Incidence rate ratios (IRRs) with 95% confidence intervals (CI) for ruck and maul injuries between backs and forwards in rugby union. Points located to the right of the vertical line suggest that the incidence of injuries is higher among forwards compared to backs. 54](#_Toc198120436)

[Supplementary figure 50: Incidence rate ratios (IRRs) with 95% confidence intervals (CI) for running injuries between backs and forwards in rugby union. Points located to the right of the vertical line suggest that the incidence of injuries is higher among forwards compared to backs. 55](#_Toc198120437)

[Supplementary figure 51: Incidence rate ratios (IRRs) with 95% confidence intervals (CI) for collision injuries between backs and forwards in rugby union. Points located to the right of the vertical line suggest that the incidence of injuries is higher among forwards compared to backs. 56](#_Toc198120438)

[Supplementary figure 52: Incidence rate ratios (IRRs) with 95% confidence intervals (CI) for medical-attention injuries between backs and forwards. Points located to the right of the vertical line suggest that the incidence of injuries is higher among forwards compared to backs. 57](#_Toc198120439)

[Supplementary figure 53: Incidence rate ratios (IRRs) with 95% confidence intervals (CI) for time-loss injuries between backs and forwards. Points located to the right of the vertical line suggest that the incidence of injuries is higher among forwards compared to backs. 58](#_Toc198120440)

[Supplementary figure 54: Funnel plot for the detection of publication bias. 59](#_Toc198120441)


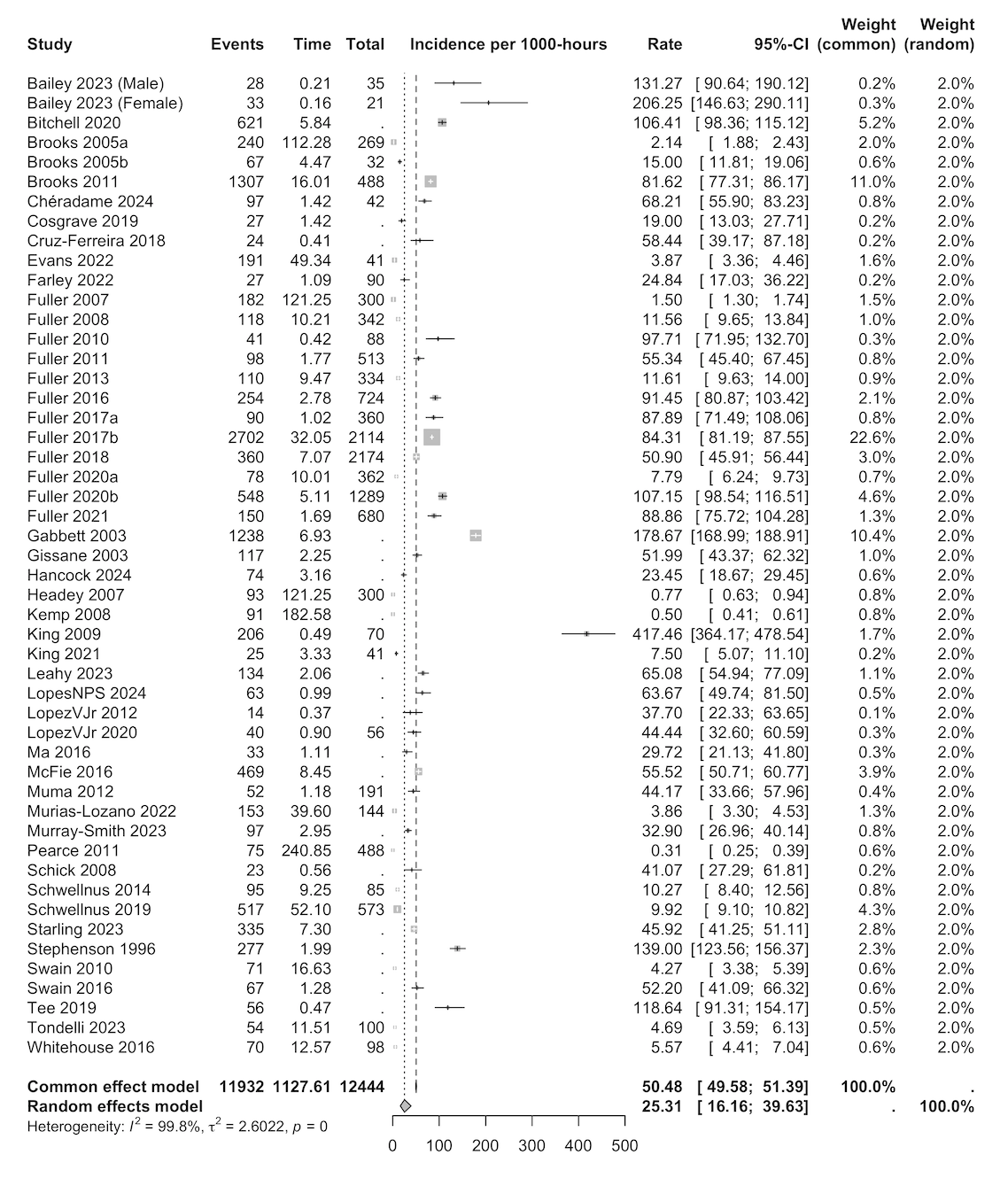


# Supplementary figure 1: The pooled injury incidence (vertical dashed line) with 95% confidence intervals (CI) (Horizontal line around the absolute incidence for each study) of rugby injuries for forwards.


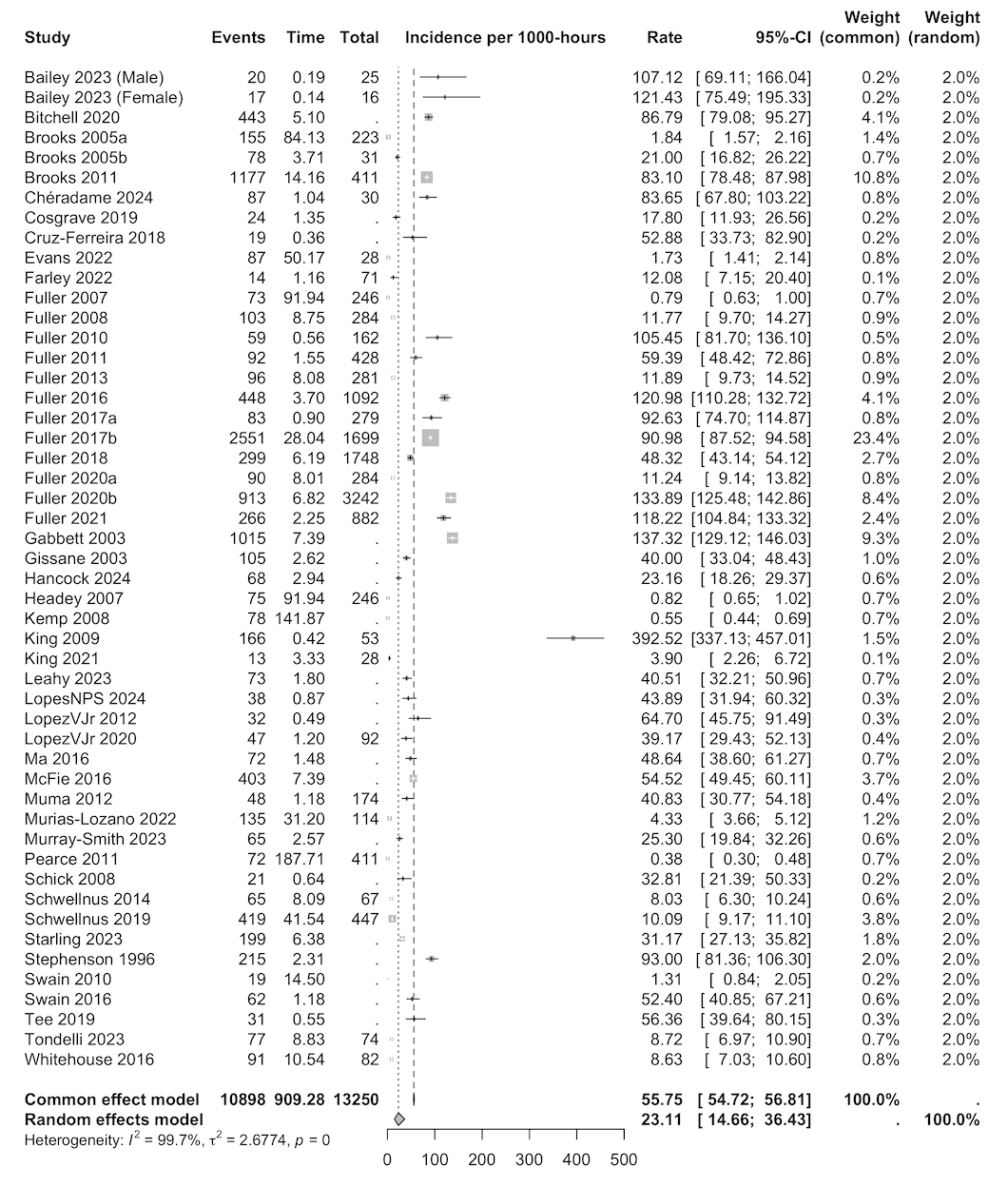


# Supplementary figure 2: The pooled injury incidence (vertical dashed line) with 95% confidence intervals (CI) (Horizontal line around the absolute incidence for each study) of rugby injuries for backs.


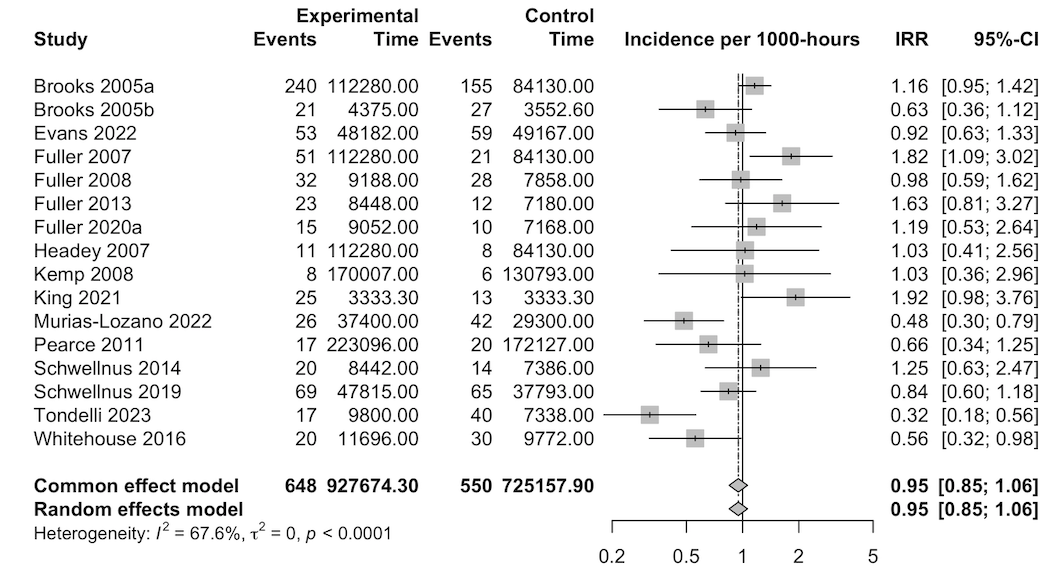


# Supplementary figure 3: Incidence rate ratios (IRRs) with 95% confidence intervals (CI) for training injuries between backs and forwards in Rugby Union. Points located to the right of the vertical line suggest that the incidence of injuries is higher among forwards compared to backs.


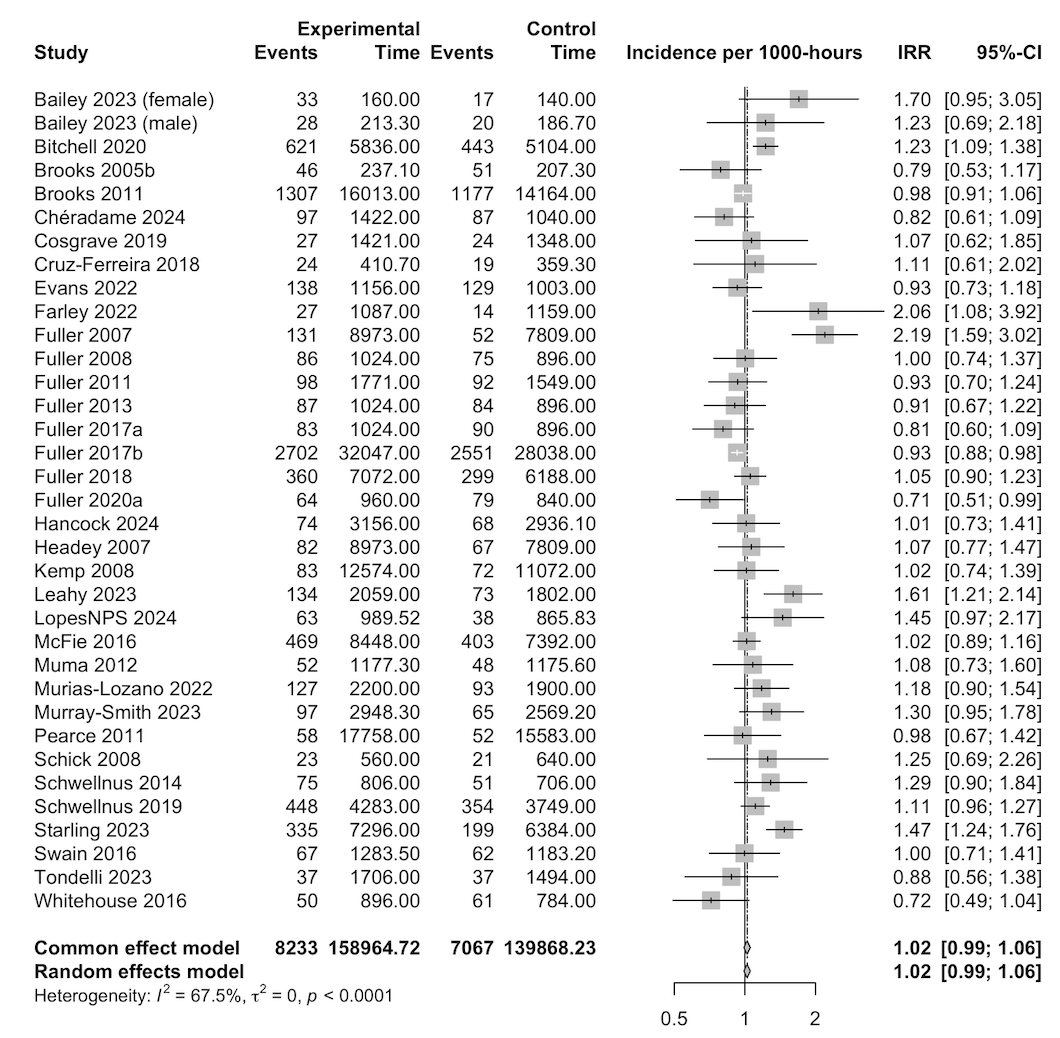


# Supplementary figure 4: Incidence rate ratios (IRRs) with 95% confidence intervals (CI) for match injuries between backs and forwards in Rugby Union. Points located to the right of the vertical line suggest that the incidence of injuries is higher among forwards compared to backs.


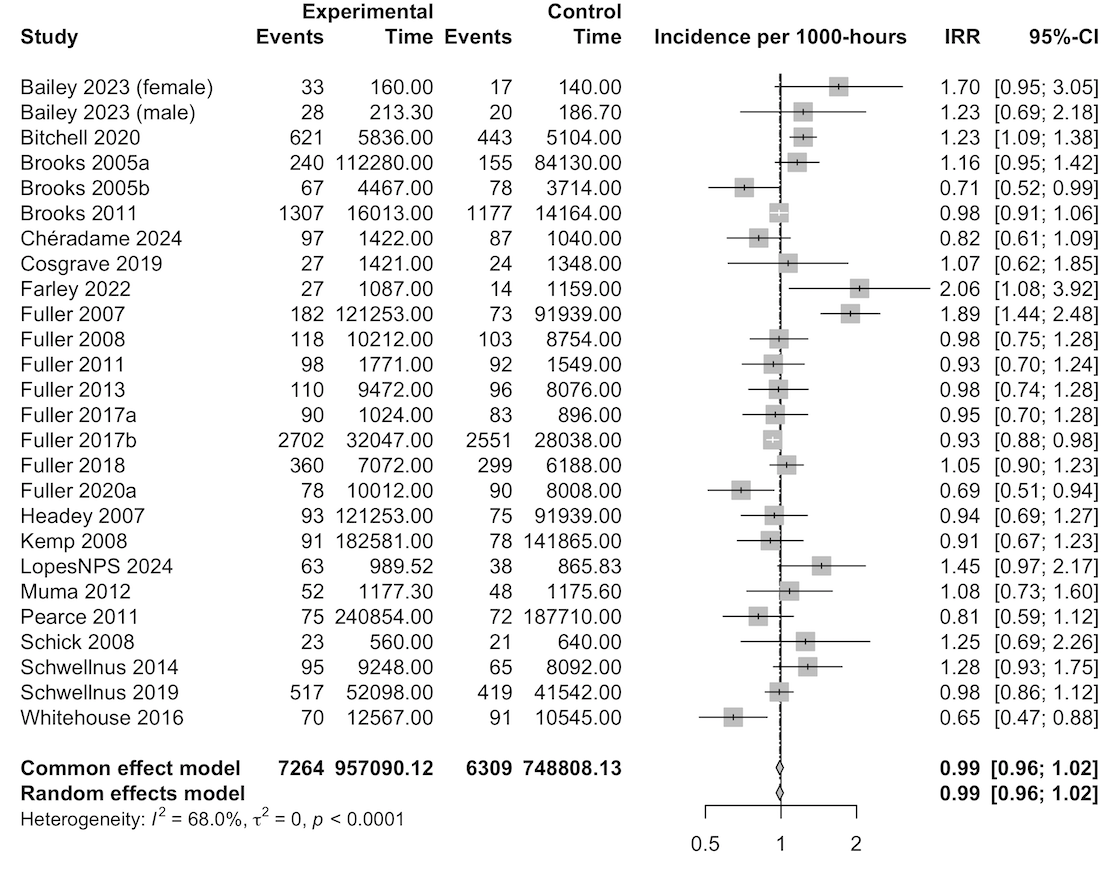


# Supplementary figure 5: Incidence rate ratios (IRRs) with 95% confidence intervals (CI) for overall injuries between professional backs and forwards in Rugby Union. Points located to the right of the vertical line suggest that the incidence of injuries is higher among forwards compared to backs.


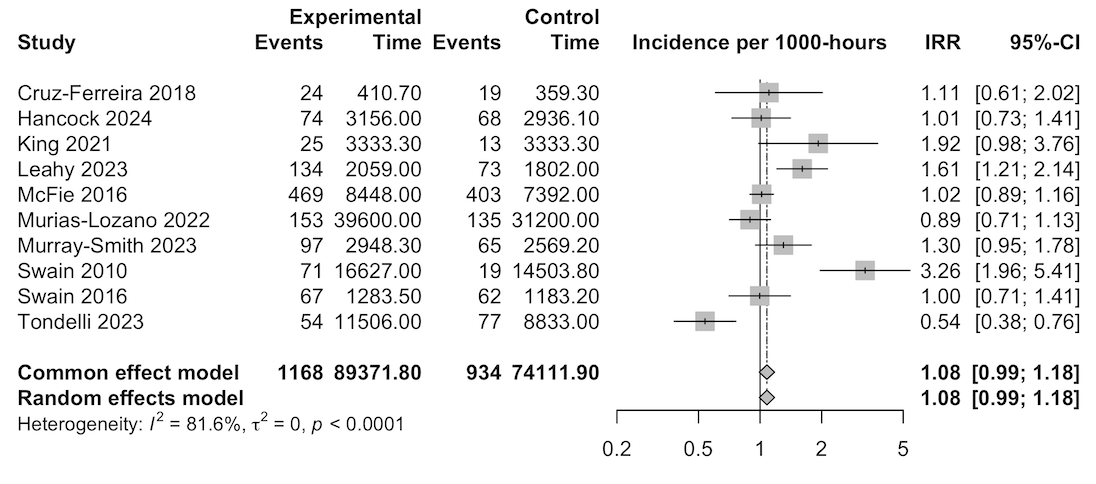


# Supplementary figure 6: Incidence rate ratios (IRRs) with 95% confidence intervals (CI) for overall injuries between amateur backs and forwards in Rugby Union. Points located to the right of the vertical line suggest that the incidence of injuries is higher among forwards compared to backs.


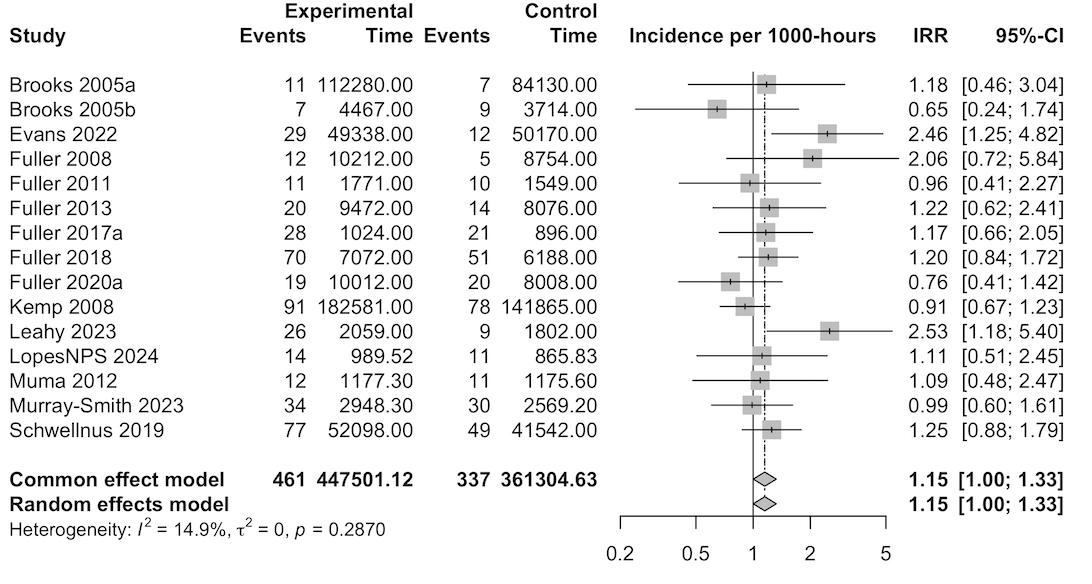


# Supplementary figure 7: Incidence rate ratios (IRRs) with 95% confidence intervals (CI) for head injuries between backs and forwards in Rugby Union. Points located to the right of the vertical line suggest that the incidence of injuries is higher among forwards compared to backs.


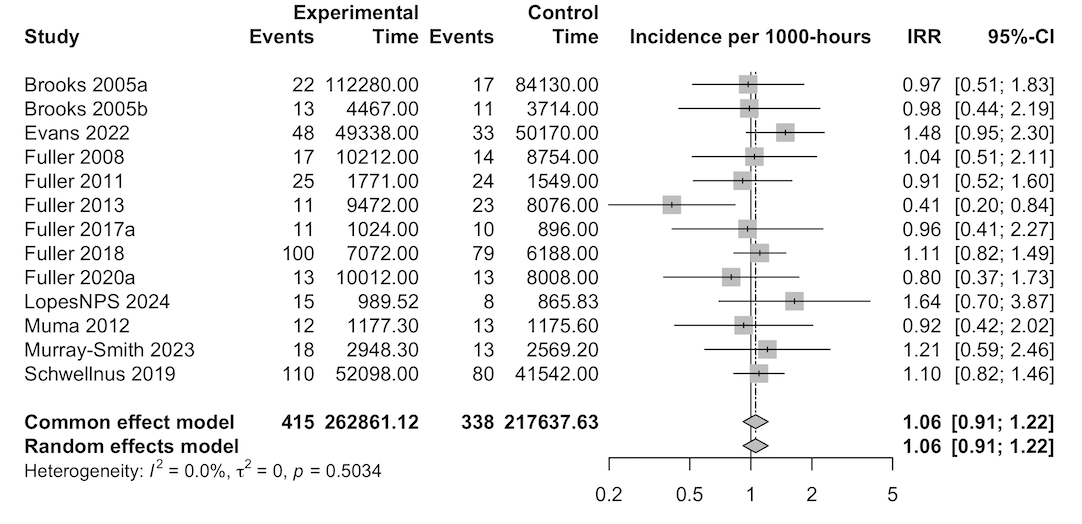


# Supplementary figure 8: Incidence rate ratios (IRRs) with 95% confidence intervals (CI) for upper limb injuries between backs and forwards in Rugby Union. Points located to the right of the vertical line suggest that the incidence of injuries is higher among forwards compared to backs.


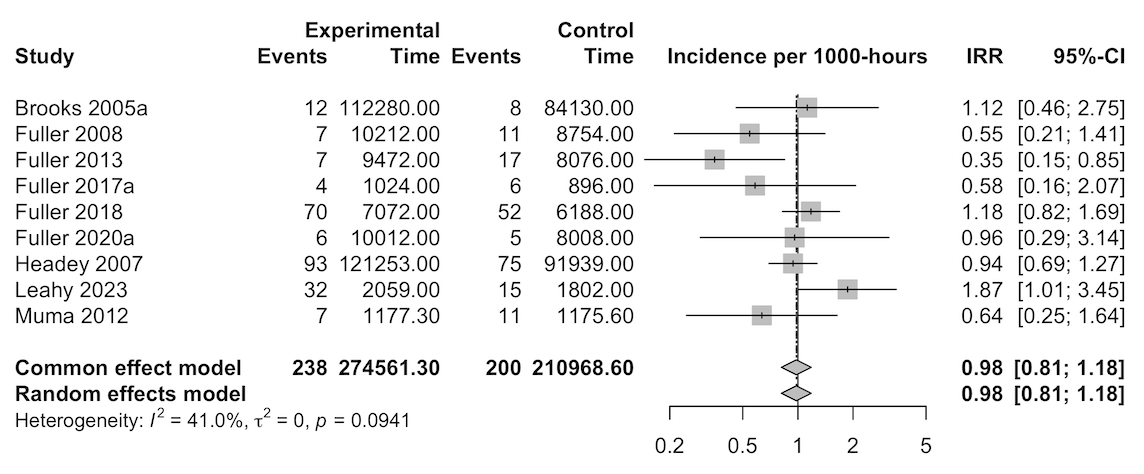


# Supplementary figure 9: Incidence rate ratios (IRRs) with 95% confidence intervals (CI) for shoulder injuries between backs and forwards in Rugby Union. Points located to the right of the vertical line suggest that the incidence of injuries is higher among forwards compared to backs.


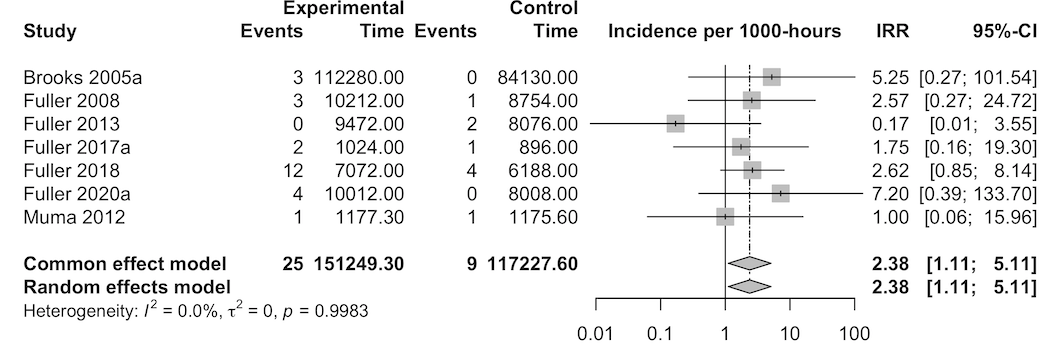


# Supplementary figure 10: Incidence rate ratios (IRRs) with 95% confidence intervals (CI) for arm and elbow injuries between backs and forwards in Rugby Union. Points located to the right of the vertical line suggest that the incidence of injuries is higher among forwards compared to backs.


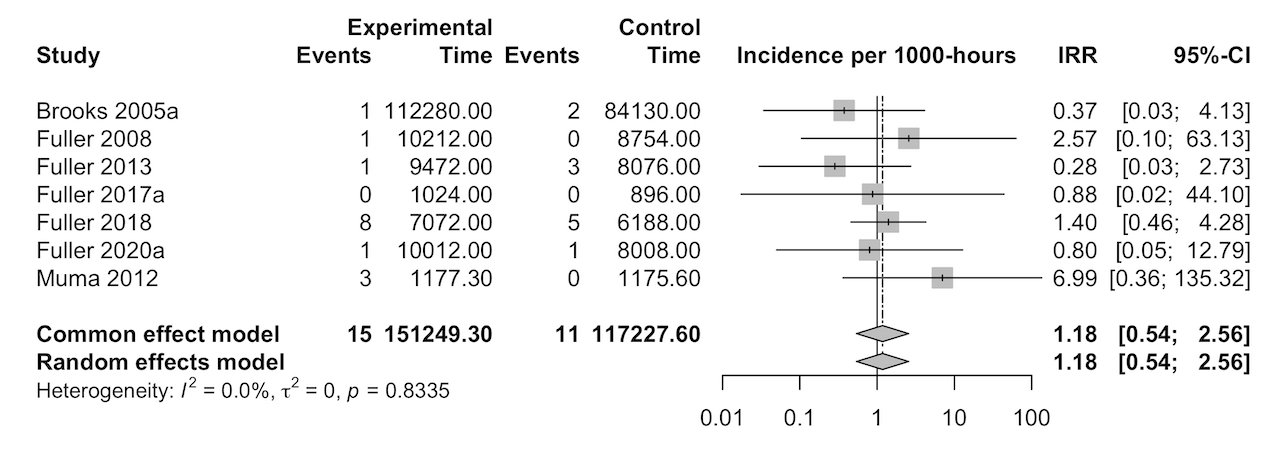


# Supplementary figure 11: Incidence rate ratios (IRRs) with 95% confidence intervals (CI) for forearm and wrist injuries between backs and forwards in Rugby Union. Points located to the right of the vertical line suggest that the incidence of injuries is higher among forwards compared to backs.


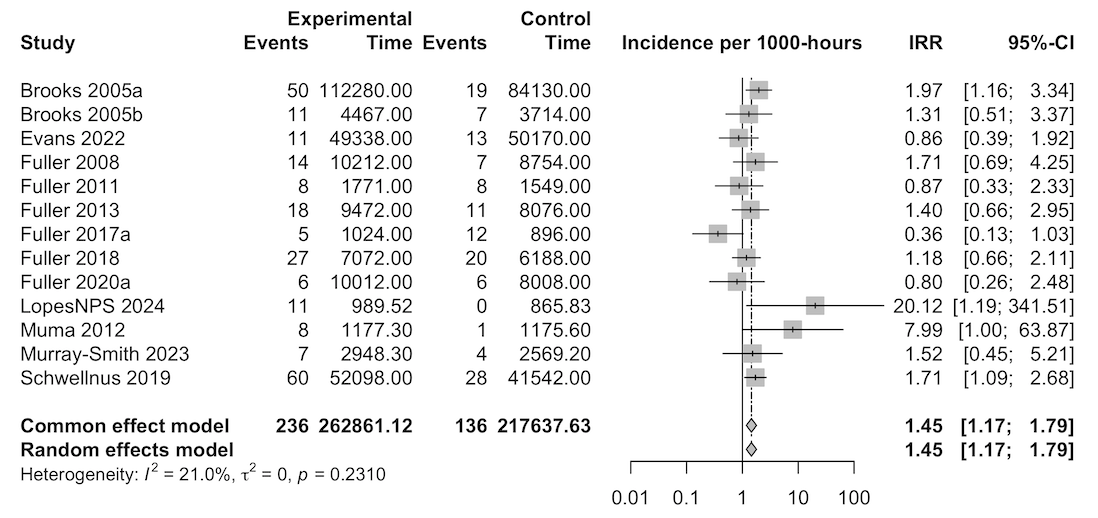


Supplementary figure 12: Incidence rate ratios (IRRs) with 95% confidence intervals (CI) for trunk and back injuries between backs and forwards in Rugby Union. Points located to the right of the vertical line suggest that the incidence of injuries is higher among forwards compared to backs.


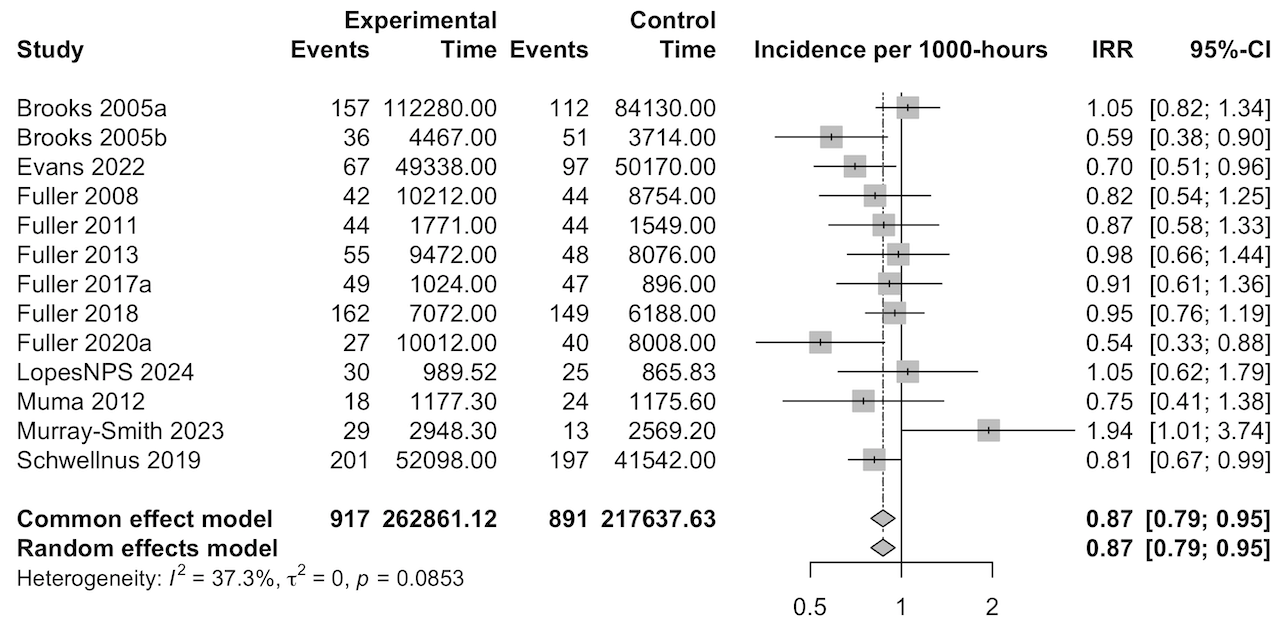


# Supplementary figure 13: Incidence rate ratios (IRRs) with 95% confidence intervals (CI) for lower limb injuries between backs and forwards in Rugby Union. Points located to the right of the vertical line suggest that the incidence of injuries is higher among forwards compared to backs.


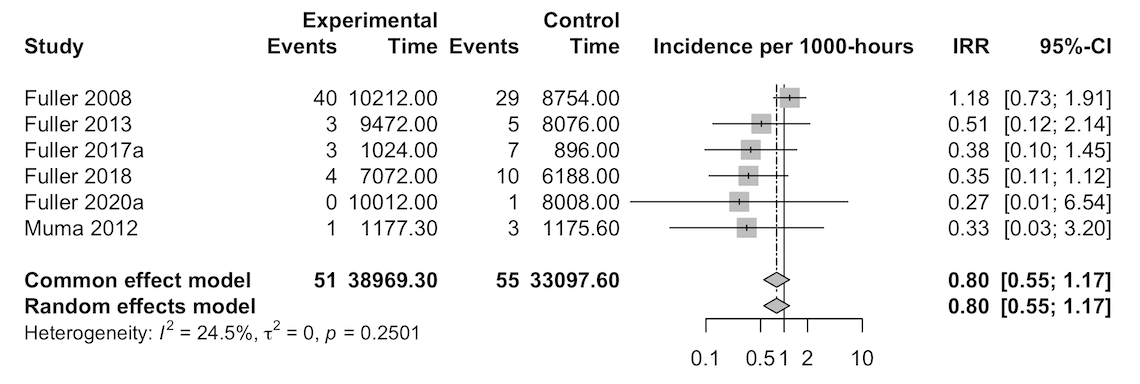


# Supplementary figure 14: Incidence rate ratios (IRRs) with 95% confidence intervals (CI) for hip and groin injuries between backs and forwards in Rugby Union. Points located to the right of the vertical line suggest that the incidence of injuries is higher among forwards compared to backs.


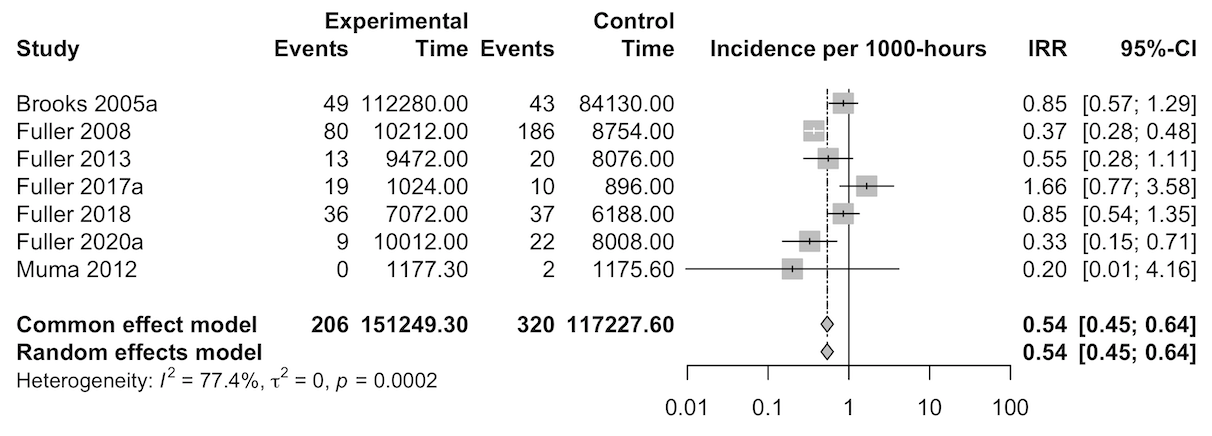


# Supplementary figure 15: Incidence rate ratios (IRRs) with 95% confidence intervals (CI) for thigh injuries between backs and forwards in Rugby Union. Points located to the right of the vertical line suggest that the incidence of injuries is higher among forwards compared to backs.


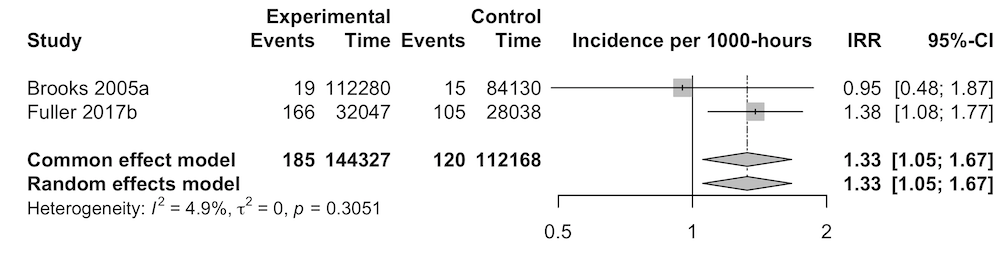


# Supplementary figure 16: Incidence rate ratios (IRRs) with 95% confidence intervals (CI) for calf injuries between backs and forwards in Rugby Union. Points located to the right of the vertical line suggest that the incidence of injuries is higher among forwards compared to backs.


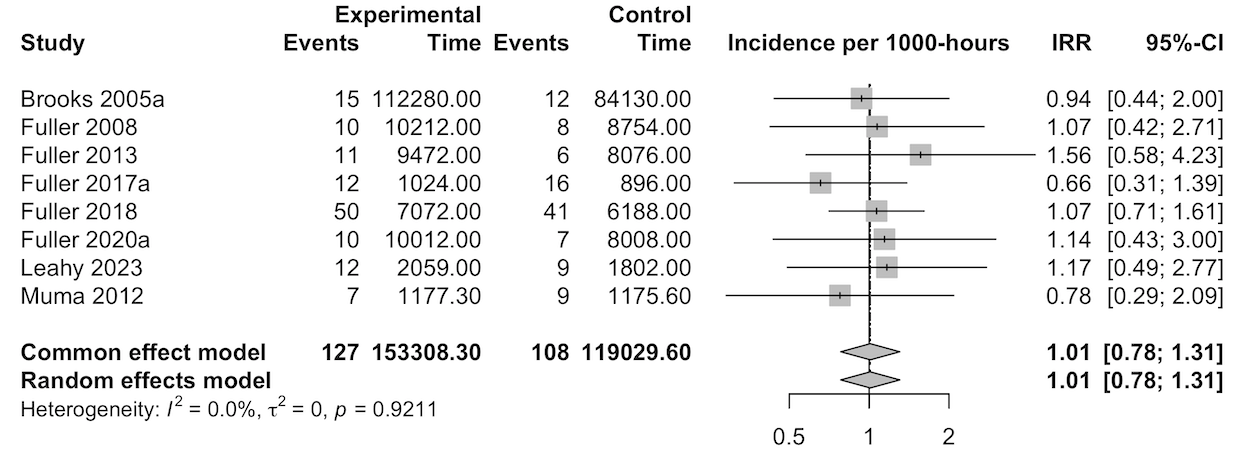


# Supplementary figure 17: Incidence rate ratios (IRRs) with 95% confidence intervals (CI) for knee injuries between backs and forwards in Rugby Union. Points located to the right of the vertical line suggest that the incidence of injuries is higher among forwards compared to backs.


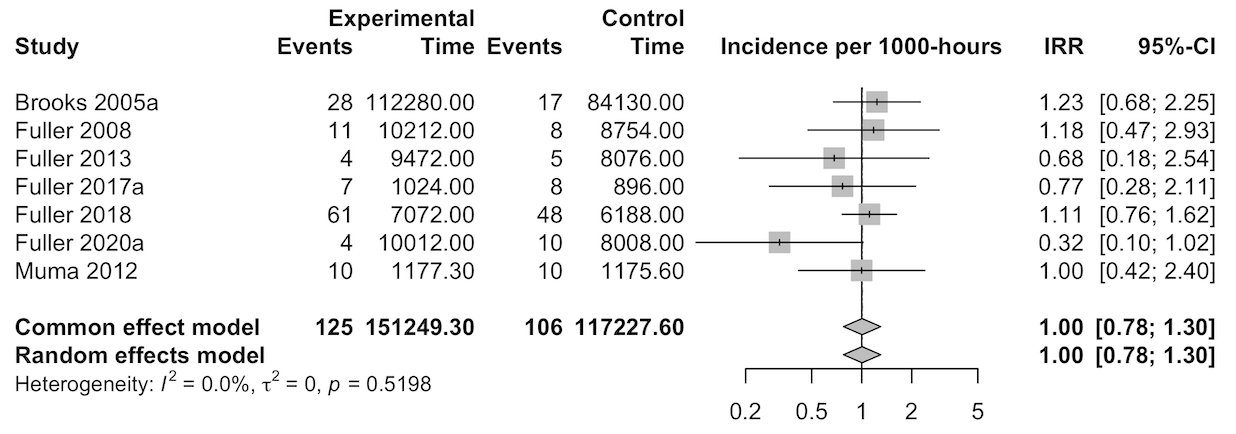


# Supplementary figure 18: Incidence rate ratios (IRRs) with 95% confidence intervals (CI) for foot and ankle injuries between backs and forwards in Rugby Union. Points located to the right of the vertical line suggest that the incidence of injuries is higher among forwards compared to backs.


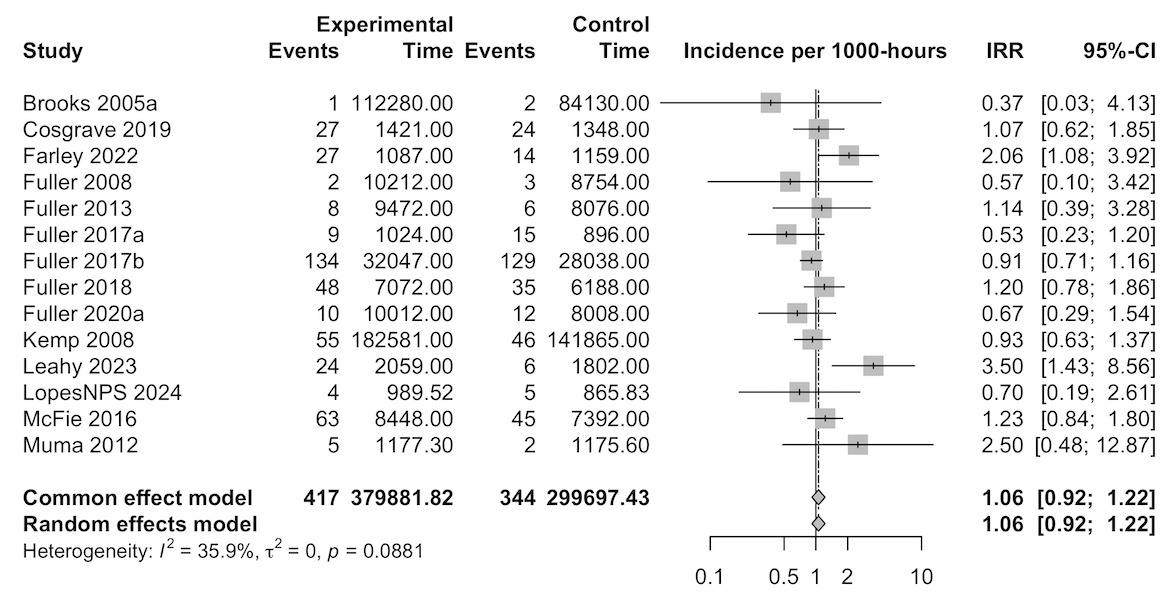


# Supplementary figure 19: Incidence rate ratios (IRRs) with 95% confidence intervals (CI) for concussions between backs and forwards in Rugby Union. Points located to the right of the vertical line suggest that the incidence of injuries is higher among forwards compared to backs.


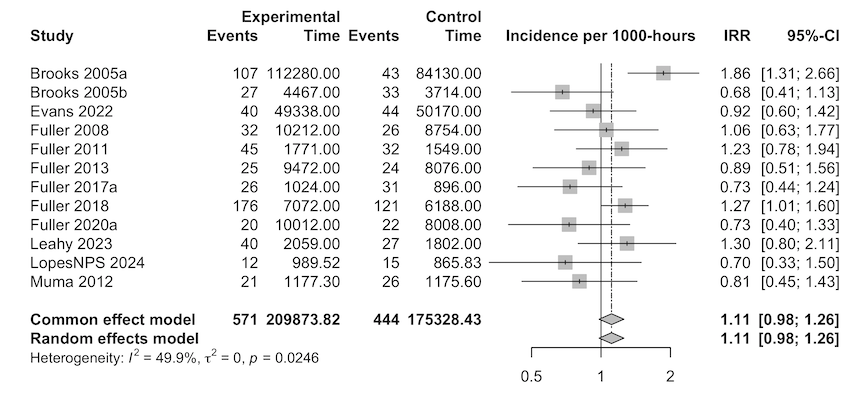


# Supplementary figure 20: Incidence rate ratios (IRRs) with 95% confidence intervals (CI) for sprain injuries between backs and forwards in Rugby Union. Points located to the right of the vertical line suggest that the incidence of injuries is higher among forwards compared to backs.


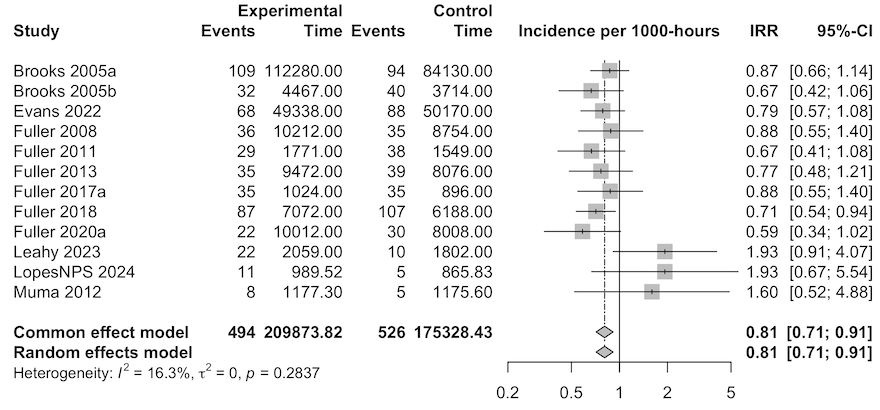


# Supplementary figure 21: Incidence rate ratios (IRRs) with 95% confidence intervals (CI) for strain injuries between backs and forwards in Rugby Union. Points located to the right of the vertical line suggest that the incidence of injuries is higher among forwards compared to backs.


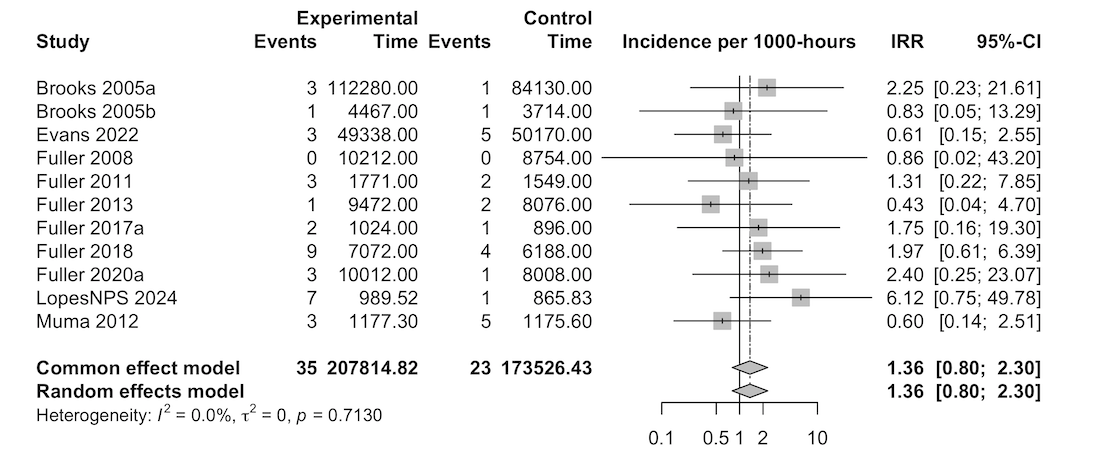


# Supplementary figure 22: Incidence rate ratios (IRRs) with 95% confidence intervals (CI) for skin laceration injuries between backs and forwards in Rugby Union. Points located to the right of the vertical line suggest that the incidence of injuries is higher among forwards compared to backs.


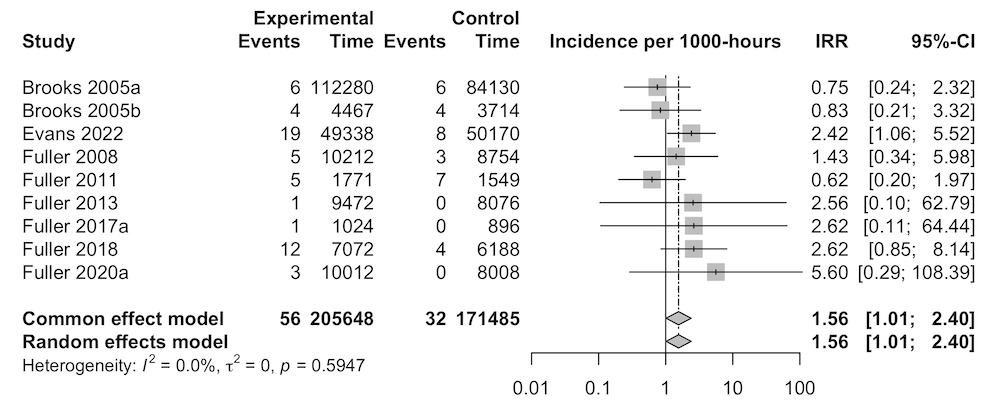


# Supplementary figure 23: Incidence rate ratios (IRRs) with 95% confidence intervals (CI) for nerve injuries (excluding concussions) between backs and forwards in Rugby Union. Points located to the right of the vertical line suggest that the incidence of injuries is higher among forwards compared to backs.


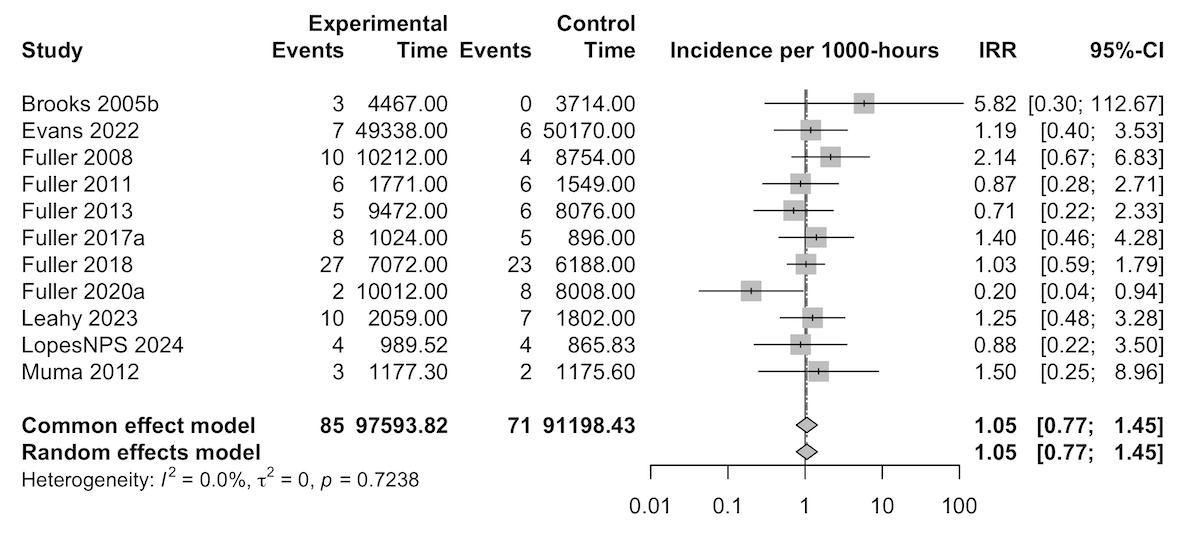


# Supplementary figure 24: Incidence rate ratios (IRRs) with 95% confidence intervals (CI) for bone injuries between backs and forwards in Rugby Union. Points located to the right of the vertical line suggest that the incidence of injuries is higher among forwards compared to backs.


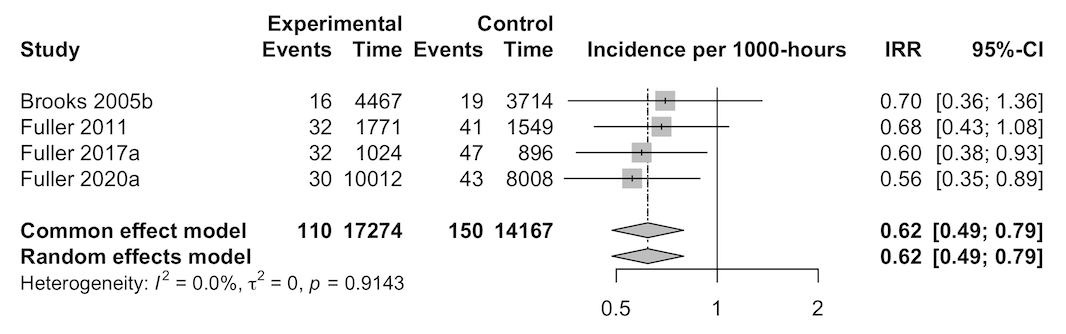


# Supplementary figure 25: Incidence rate ratios (IRRs) with 95% confidence intervals (CI) for tackle injuries between backs and forwards in Rugby Union. Points located to the right of the vertical line suggest that the incidence of injuries is higher among forwards compared to backs.


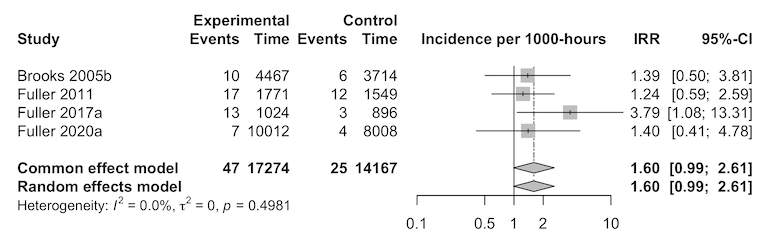


# Supplementary figure 26: Incidence rate ratios (IRRs) with 95% confidence intervals (CI) for ruck and maul injuries between backs and forwards in Rugby Union. Points located to the right of the vertical line suggest that the incidence of injuries is higher among forwards compared to backs.


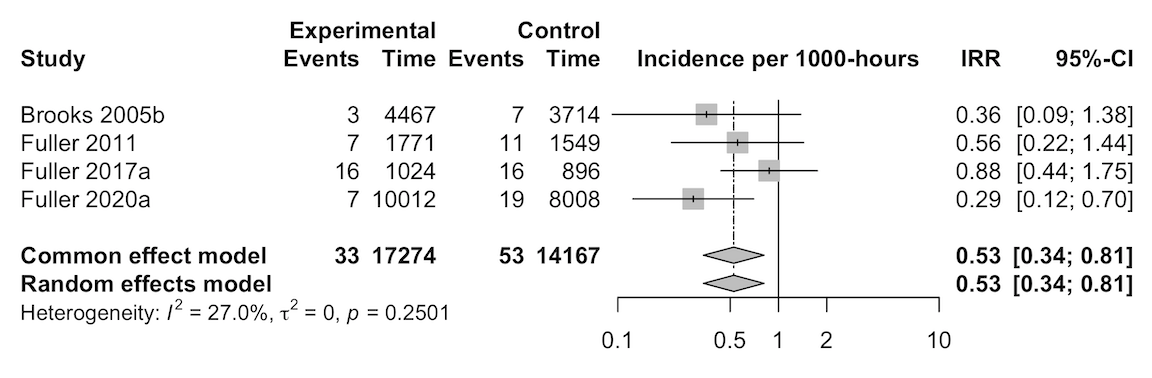


# Supplementary figure 27: Incidence rate ratios (IRRs) with 95% confidence intervals (CI) for running injuries between backs and forwards in Rugby Union. Points located to the right of the vertical line suggest that the incidence of injuries is higher among forwards compared to backs.


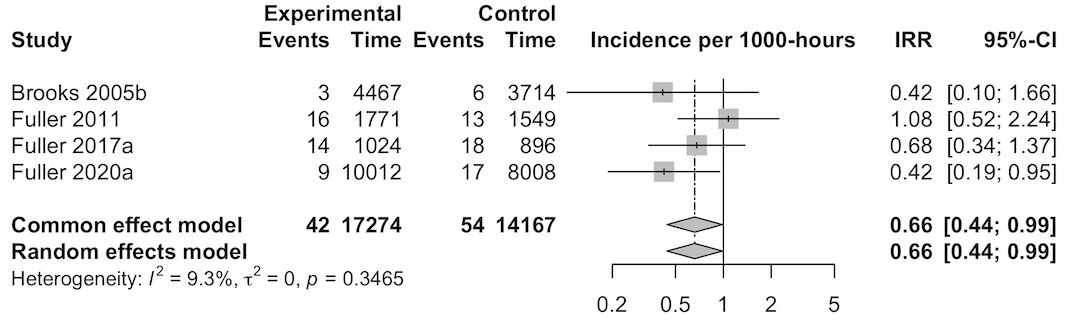


# Supplementary figure 28: Incidence rate ratios (IRRs) with 95% confidence intervals (CI) for collision injuries between backs and forwards in Rugby Union. Points located to the right of the vertical line suggest that the incidence of injuries is higher among forwards compared to backs.


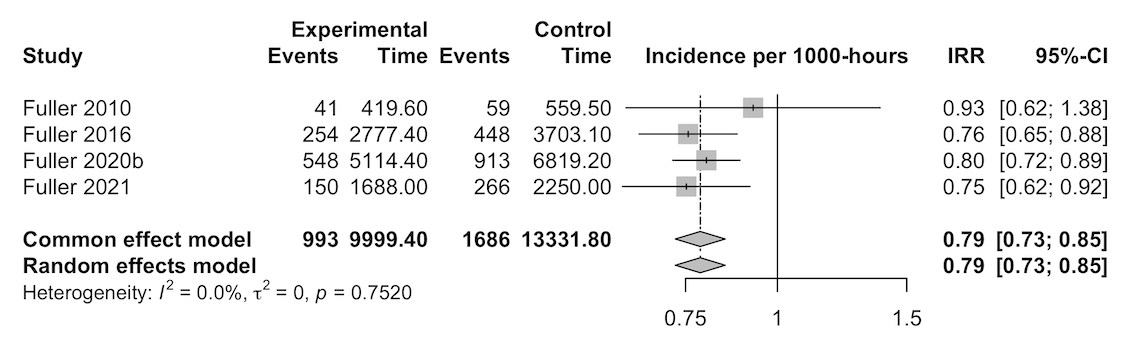


# Supplementary figure 29: Incidence rate ratios (IRRs) with 95% confidence intervals (CI) for overall injuries between professional backs and forwards in Rugby Sevens. Points located to the right of the vertical line suggest that the incidence of injuries is higher among forwards compared to backs.


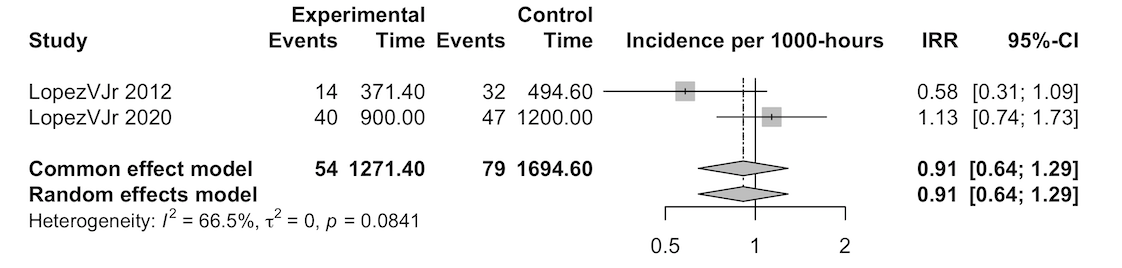


# Supplementary figure 30: Incidence rate ratios (IRRs) with 95% confidence intervals (CI) for overall injuries between amateur backs and forwards in Rugby Sevens. Points located to the right of the vertical line suggest that the incidence of injuries is higher among forwards compared to backs.


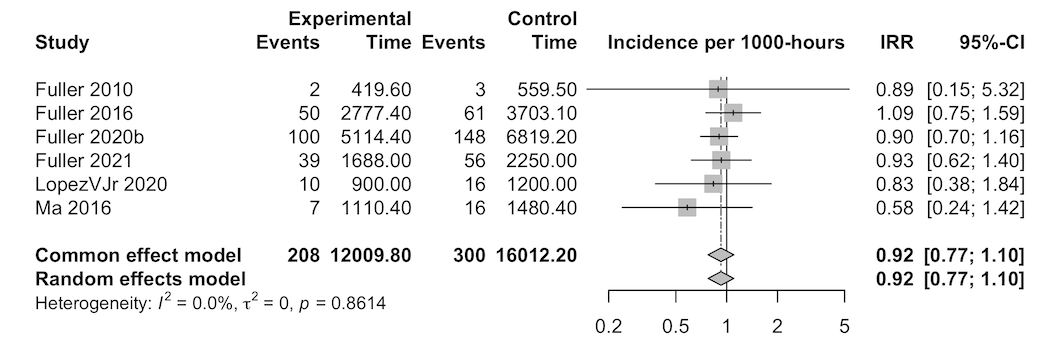


# Supplementary figure 31: Incidence rate ratios (IRRs) with 95% confidence intervals (CI) for head injuries between backs and forwards in Rugby Sevens. Points located to the right of the vertical line suggest that the incidence of injuries is higher among forwards compared to backs.


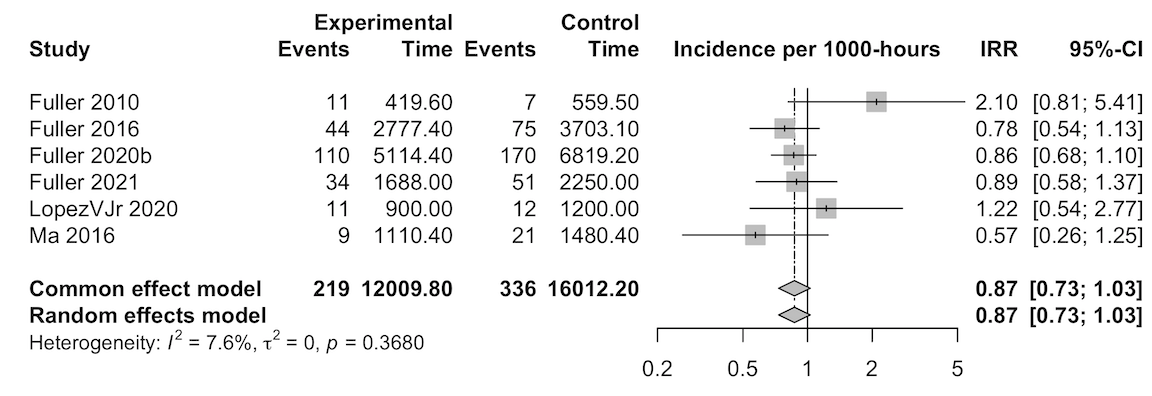


# Supplementary figure 32: Incidence rate ratios (IRRs) with 95% confidence intervals (CI) for upper limb injuries between backs and forwards in Rugby Sevens. Points located to the right of the vertical line suggest that the incidence of injuries is higher among forwards compared to backs.


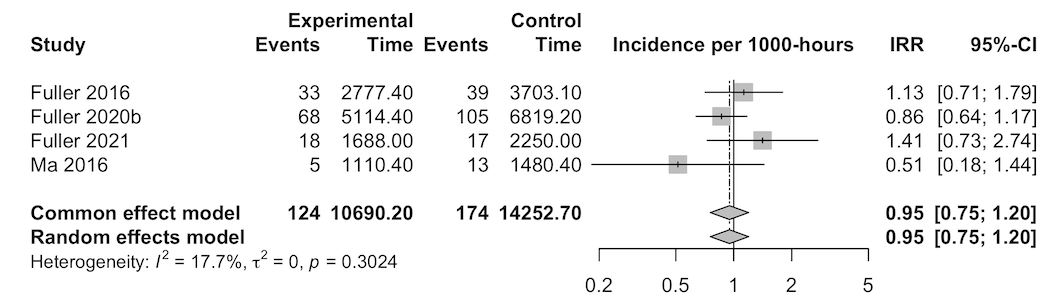


# Supplementary figure 33: Incidence rate ratios (IRRs) with 95% confidence intervals (CI) for shoulder injuries between backs and forwards in Rugby Sevens. Points located to the right of the vertical line suggest that the incidence of injuries is higher among forwards compared to backs.


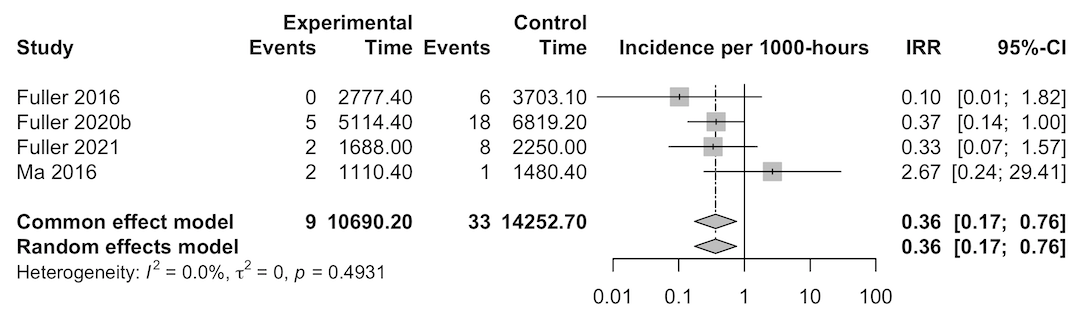


# Supplementary figure 34: Incidence rate ratios (IRRs) with 95% confidence intervals (CI) for arm and elbow injuries between backs and forwards in Rugby Sevens. Points located to the right of the vertical line suggest that the incidence of injuries is higher among forwards compared to backs.


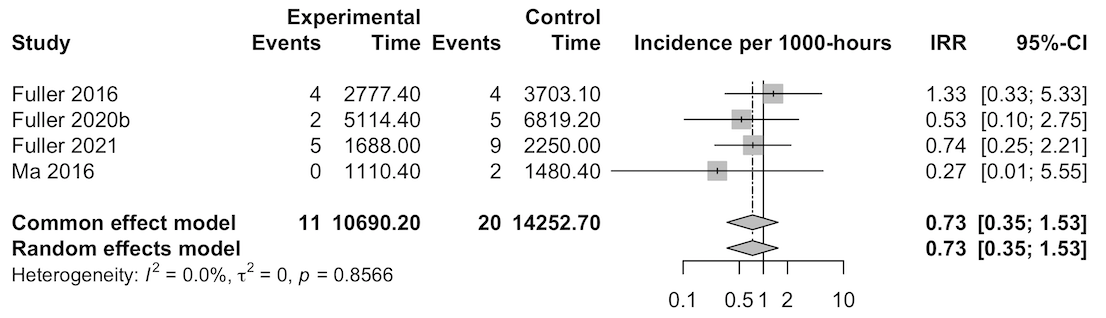


# Supplementary figure 35: Incidence rate ratios (IRRs) with 95% confidence intervals (CI) for forearm and wrist injuries between backs and forwards in Rugby Sevens. Points located to the right of the vertical line suggest that the incidence of injuries is higher among forwards compared to backs.


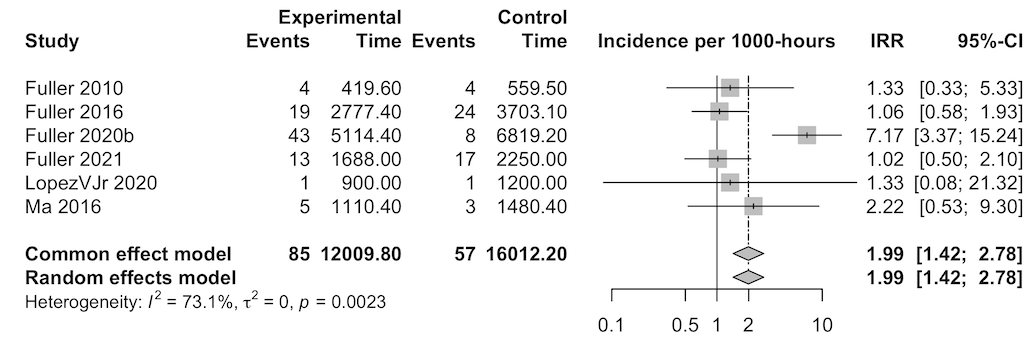


# Supplementary figure 36: Incidence rate ratios (IRRs) with 95% confidence intervals (CI) for trunk and back injuries between backs and forwards in Rugby Sevens. Points located to the right of the vertical line suggest that the incidence of injuries is higher among forwards compared to backs.


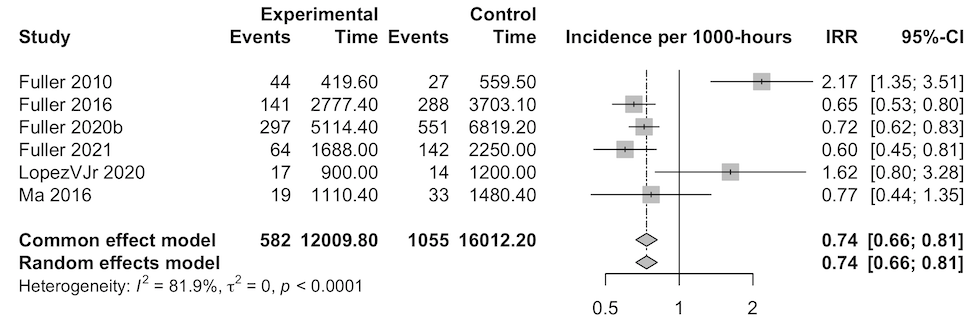


# Supplementary figure 37: Incidence rate ratios (IRRs) with 95% confidence intervals (CI) for lower limb injuries between backs and forwards in Rugby Sevens. Points located to the right of the vertical line suggest that the incidence of injuries is higher among forwards compared to backs.


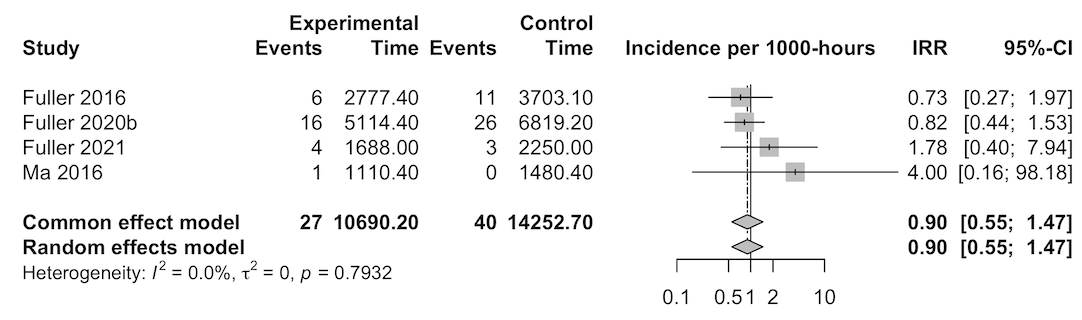


# Supplementary figure 38: Incidence rate ratios (IRRs) with 95% confidence intervals (CI) for hip and groin injuries between backs and forwards in Rugby Sevens. Points located to the right of the vertical line suggest that the incidence of injuries is higher among forwards compared to backs.


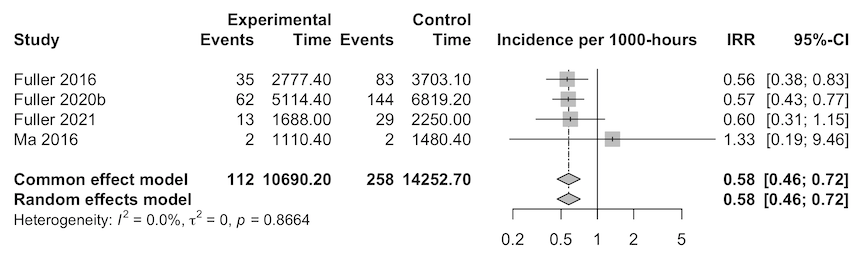


# Supplementary figure 39: Incidence rate ratios (IRRs) with 95% confidence intervals (CI) for thigh injuries between backs and forwards in Rugby Sevens. Points located to the right of the vertical line suggest that the incidence of injuries is higher among forwards compared to backs.


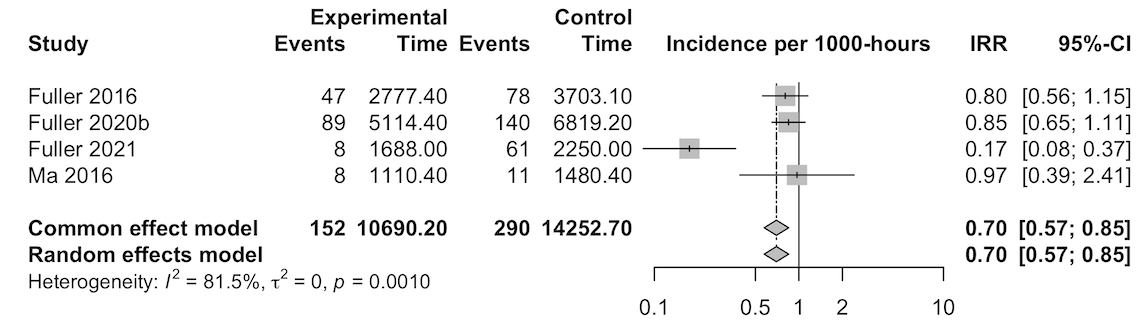


# Supplementary figure 40: Incidence rate ratios (IRRs) with 95% confidence intervals (CI) for knee injuries between backs and forwards in Rugby Sevens. Points located to the right of the vertical line suggest that the incidence of injuries is higher among forwards compared to backs.


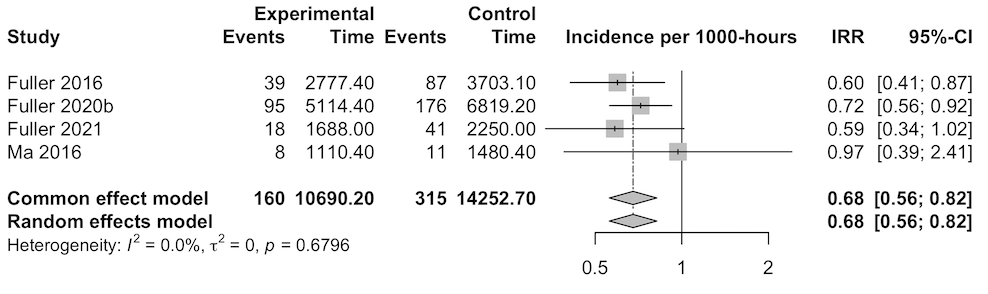


# Supplementary figure 41: Incidence rate ratios (IRRs) with 95% confidence intervals (CI) for foot and ankle injuries between backs and forwards in Rugby Sevens. Points located to the right of the vertical line suggest that the incidence of injuries is higher among forwards compared to backs.


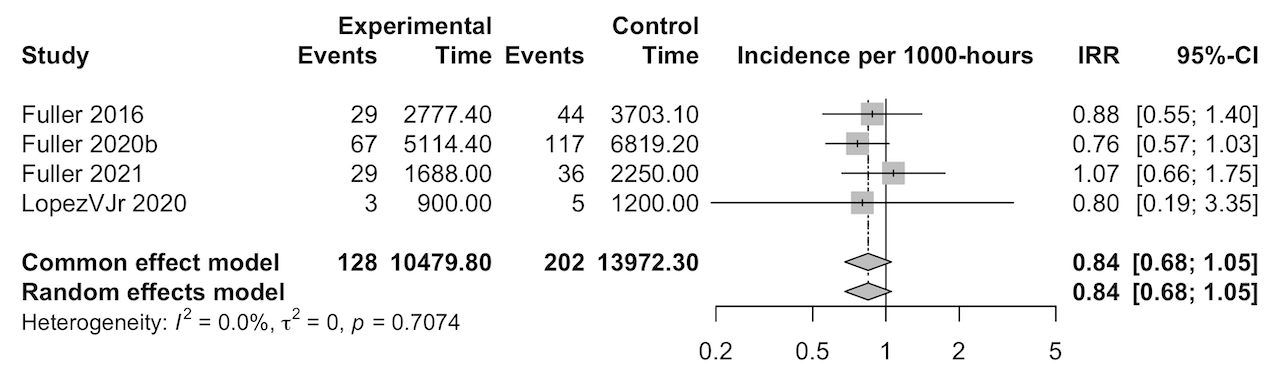


# Supplementary figure 42: Incidence rate ratios (IRRs) with 95% confidence intervals (CI) for concussions between backs and forwards in Rugby Sevens. Points located to the right of the vertical line suggest that the incidence of injuries is higher among forwards compared to backs.


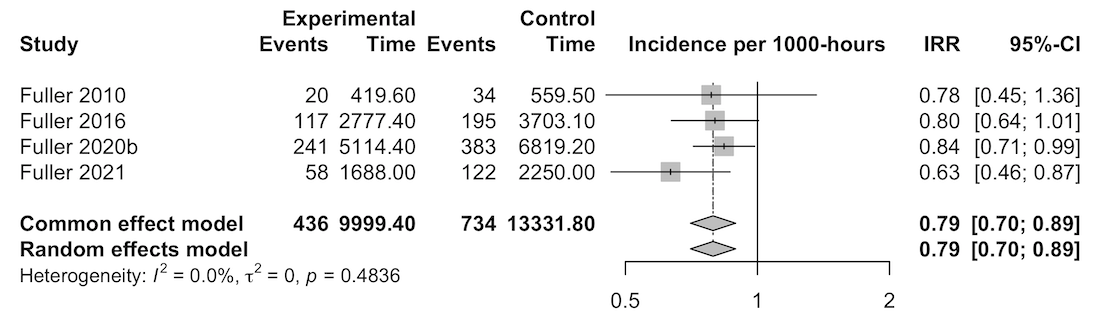


# Supplementary figure 43: Incidence rate ratios (IRRs) with 95% confidence intervals (CI) for sprain injuries between backs and forwards in Rugby Sevens. Points located to the right of the vertical line suggest that the incidence of injuries is higher among forwards compared to backs.


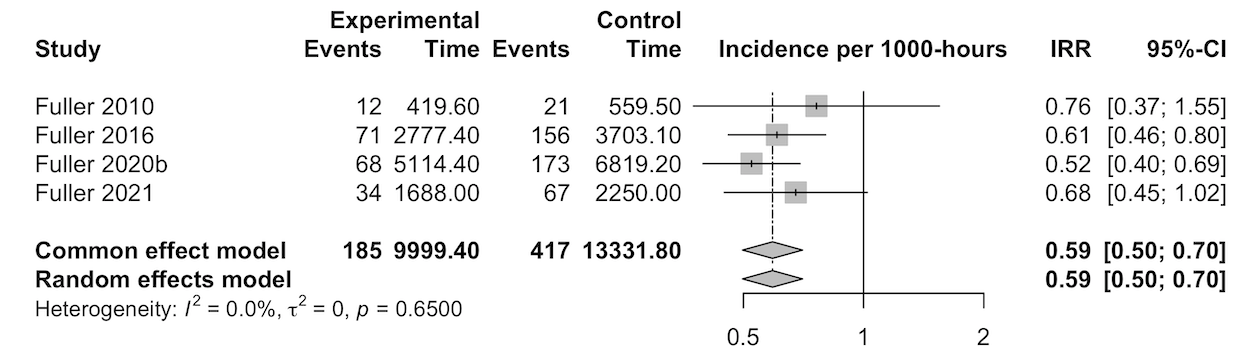


# Supplementary figure 44: Incidence rate ratios (IRRs) with 95% confidence intervals (CI) for strain injuries between backs and forwards in Rugby Sevens. Points located to the right of the vertical line suggest that the incidence of injuries is higher among forwards compared to backs.


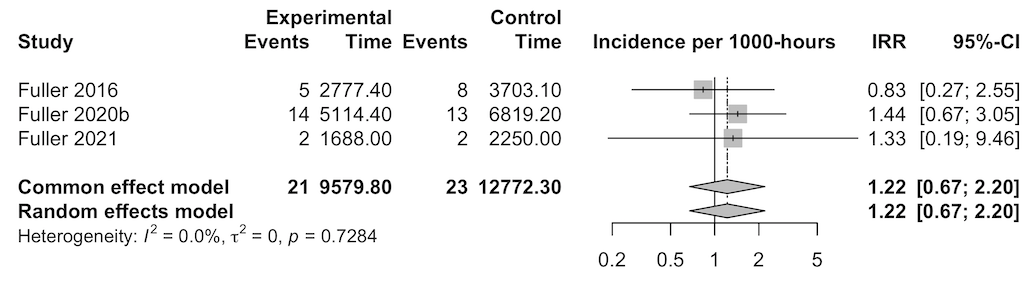


# Supplementary figure 45: Incidence rate ratios (IRRs) with 95% confidence intervals (CI) for skin laceration injuries between backs and forwards in Rugby Sevens. Points located to the right of the vertical line suggest that the incidence of injuries is higher among forwards compared to backs.


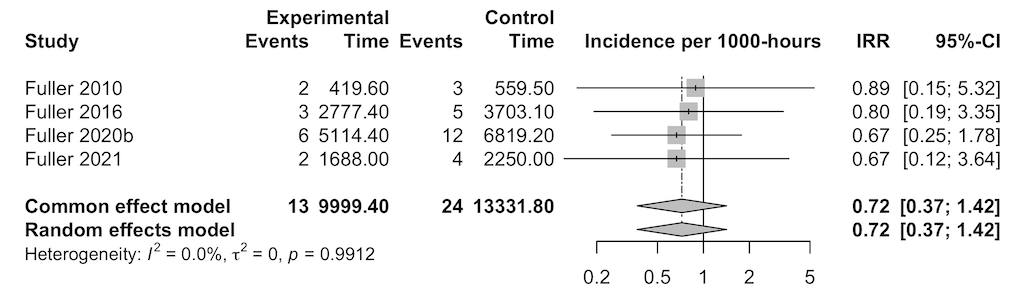


# Supplementary figure 46: Incidence rate ratios (IRRs) with 95% confidence intervals (CI) for nerve injuries (excluding concussions) between backs and forwards in Rugby Sevens. Points located to the right of the vertical line suggest that the incidence of injuries is higher among forwards compared to backs.


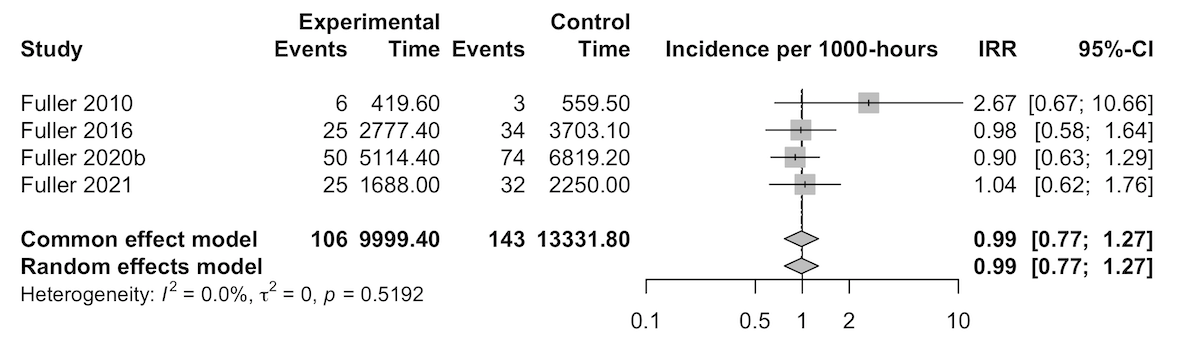


# Supplementary figure 47: Incidence rate ratios (IRRs) with 95% confidence intervals (CI) for bone injuries between backs and forwards in Rugby Sevens. Points located to the right of the vertical line suggest that the incidence of injuries is higher among forwards compared to backs.


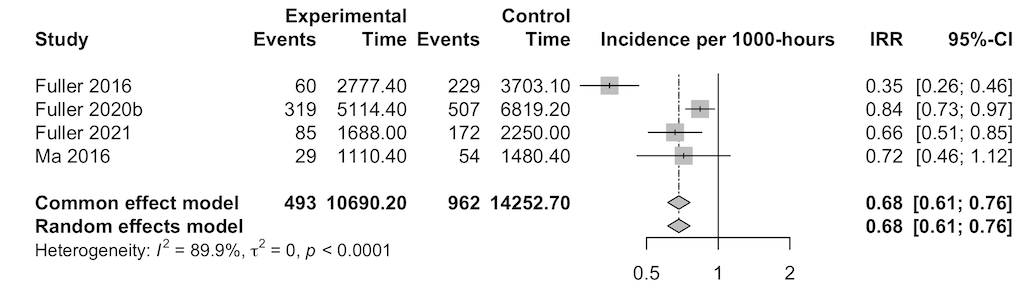


# Supplementary figure 48: Incidence rate ratios (IRRs) with 95% confidence intervals (CI) for tackle injuries between backs and forwards in Rugby Sevens. Points located to the right of the vertical line suggest that the incidence of injuries is higher among forwards compared to backs.


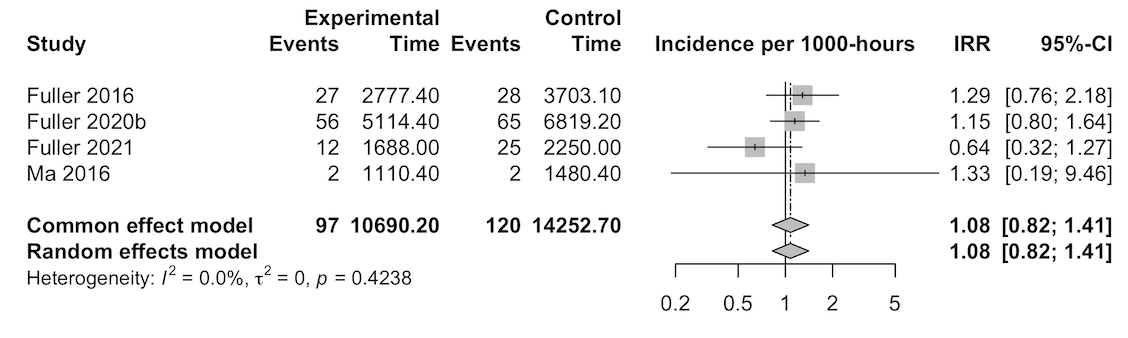


# Supplementary figure 49: Incidence rate ratios (IRRs) with 95% confidence intervals (CI) for ruck and maul injuries between backs and forwards in Rugby Sevens. Points located to the right of the vertical line suggest that the incidence of injuries is higher among forwards compared to backs.


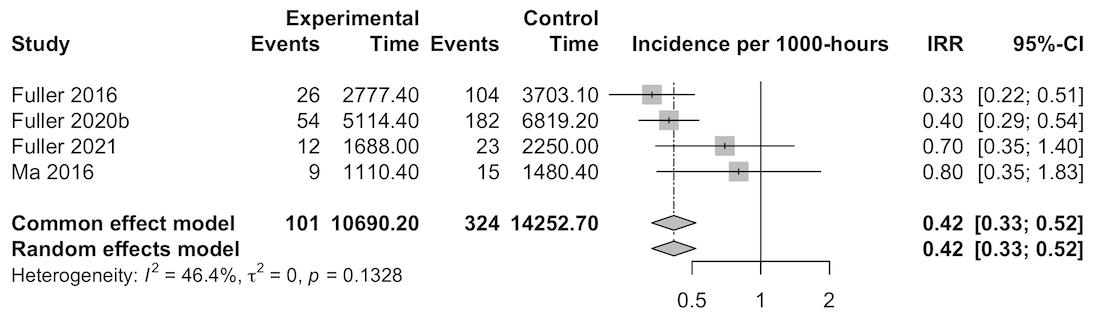


# Supplementary figure 50: Incidence rate ratios (IRRs) with 95% confidence intervals (CI) for running injuries between backs and forwards in Rugby Sevens. Points located to the right of the vertical line suggest that the incidence of injuries is higher among forwards compared to backs.


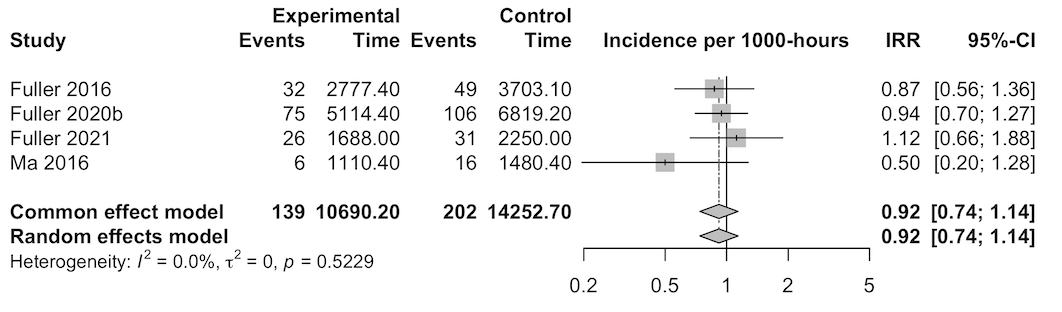


# Supplementary figure 51: Incidence rate ratios (IRRs) with 95% confidence intervals (CI) for collision injuries between backs and forwards in Rugby Sevens. Points located to the right of the vertical line suggest that the incidence of injuries is higher among forwards compared to backs.


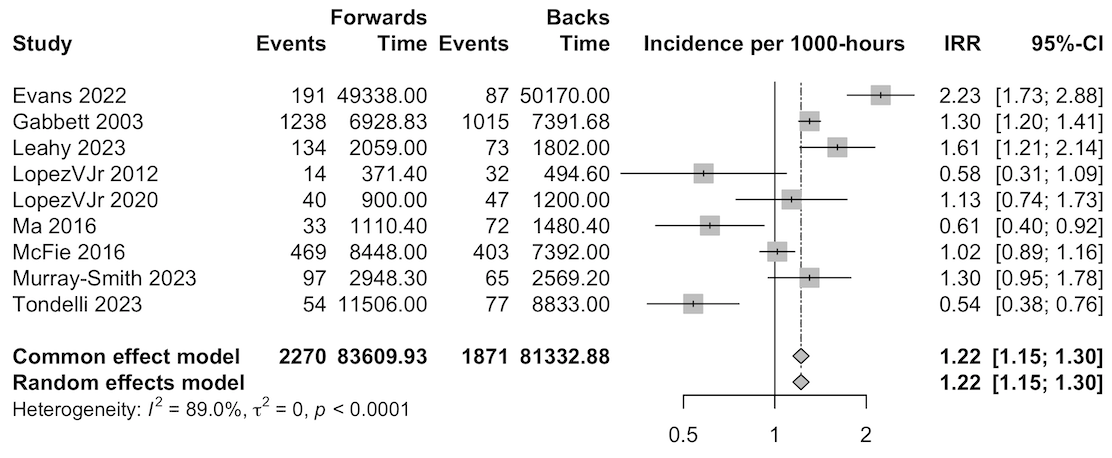


# Supplementary figure 52: Incidence rate ratios (IRRs) with 95% confidence intervals (CI) for medical-attention injuries between backs and forwards. Points located to the right of the vertical line suggest that the incidence of injuries is higher among forwards compared to backs.


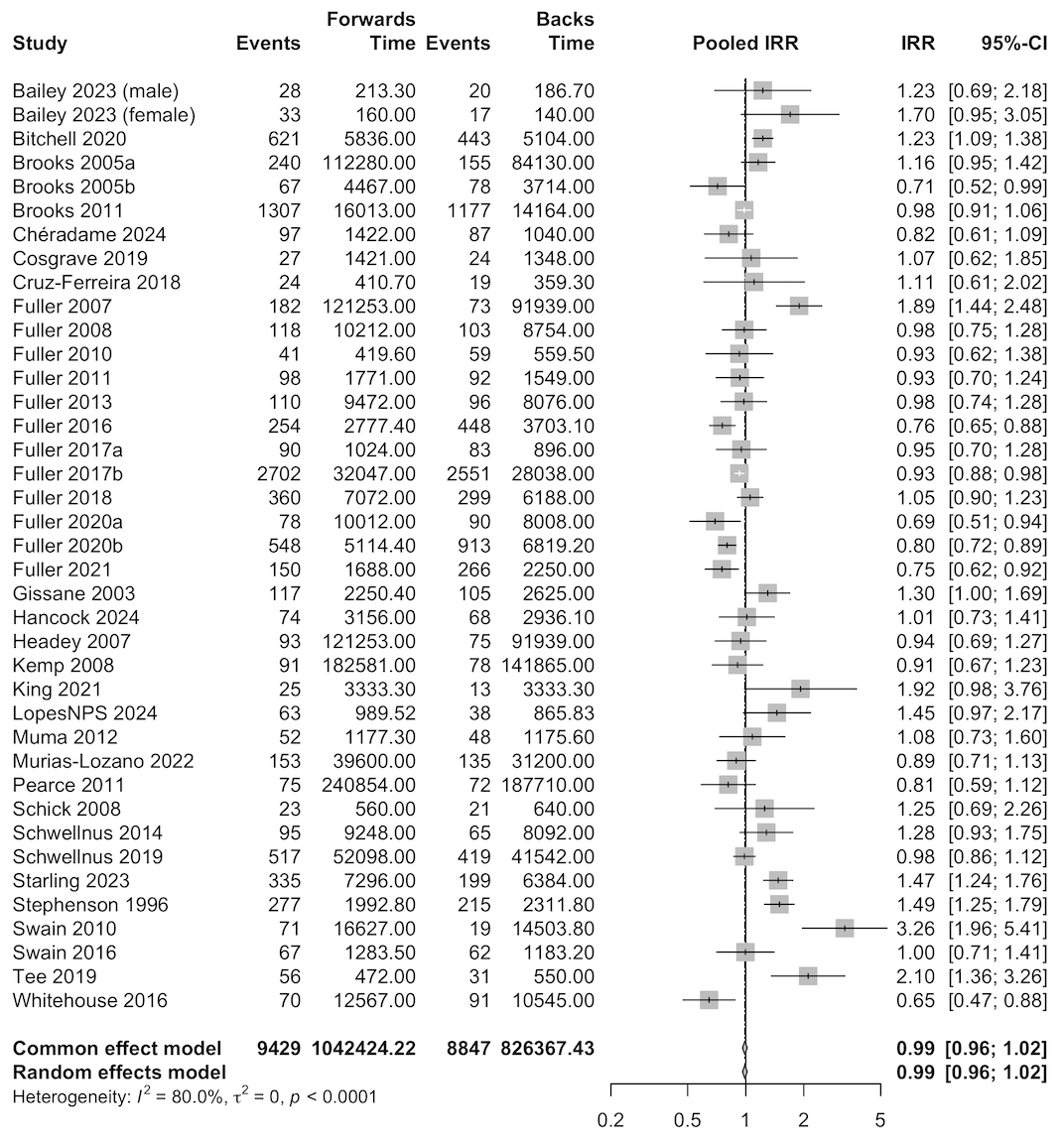


# Supplementary figure 53: Incidence rate ratios (IRRs) with 95% confidence intervals (CI) for time-loss injuries between backs and forwards. Points located to the right of the vertical line suggest that the incidence of injuries is higher among forwards compared to backs.


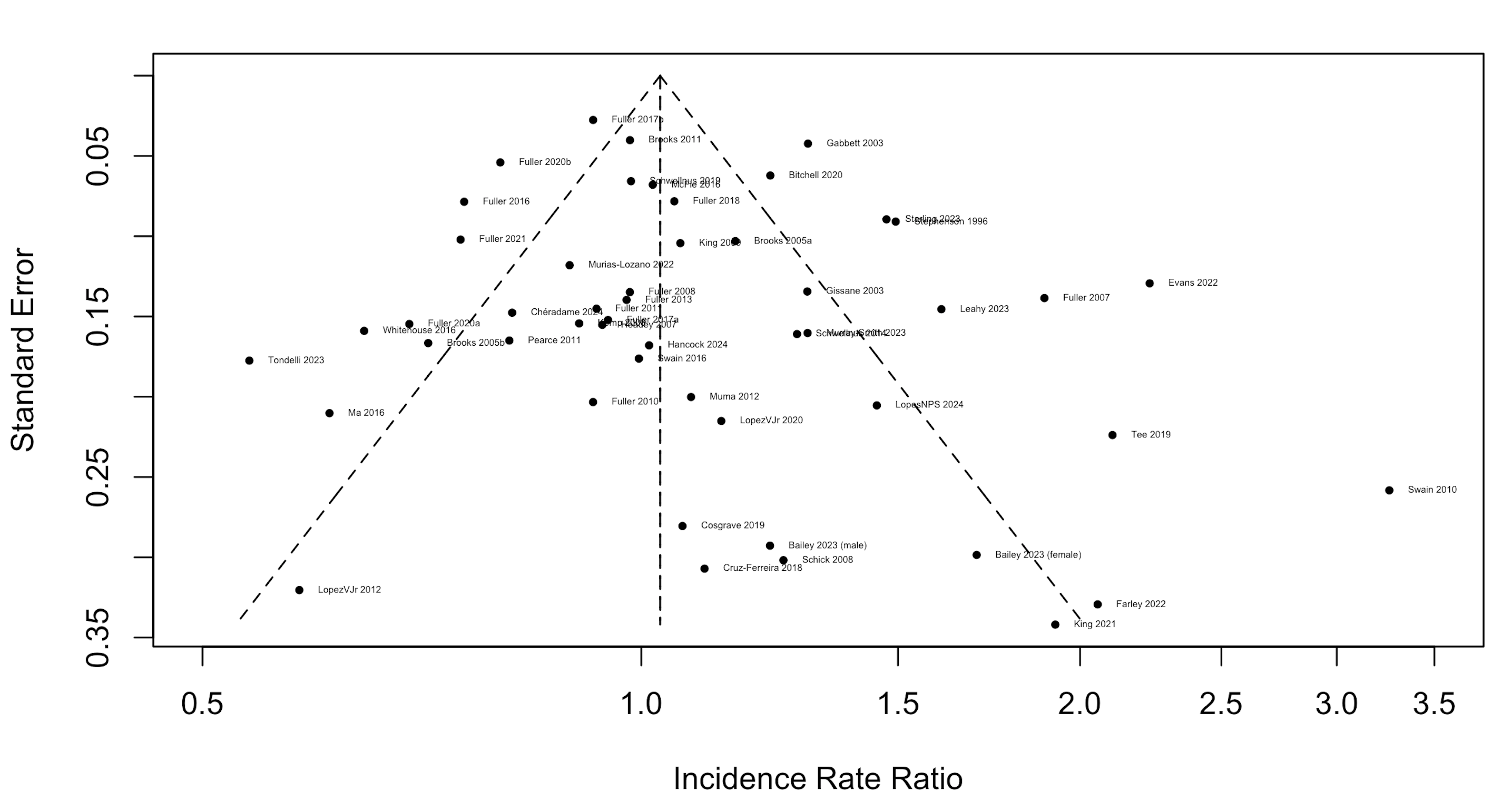


# Supplementary figure 54: Funnel plot for the detection of publication bias.
