# Supplementary material for: Differences in Injury Incidence Between Player Positions Across All Rugby Formats—A Systematic Review and Meta‐Analysis
Source: Scand J Med Sci Sports. 2025 Jul 7;35(7):e70102. doi: 10.1111/sms.70102 (PMC12233056; doi:10.1111/sms.70102)
Supplement: Supplementary file 4 — Appendix S4. [file SMS-35-e70102-s001.docx]

Supplementary table 1: Characteristics of the included studies.

| **Study** | **Format** | **Sex** | **Age** | **Level** | **Study period** | **Country** | **Study design** | **Play** | **Injury definition** | **Injury types** |
| --- | --- | --- | --- | --- | --- | --- | --- | --- | --- | --- |
| Bailey 2023 ^18^ | Union | Both | Adult | Professional | 2017-2019 | Scotland | Prospective | Match | Time-loss | All injuries |
| Bitchell 2020 ^19^ | Union | Male | Adult | Professional | 2013-2016 | Wales | Prospective | Match | Time-loss | All injuries |
| Brooks 2005 ^20^ | Union | Both | Adult | Professional | 2002-2004 | United Kingdom | Prospective | Training | Time-loss | All injuries |
| Brooks 2005 ^21^ | Union | Male | Adult | Professional | 2002-2003 | United Kingdom | Prospective | Both | Time-loss | All injuries |
| Brooks 2011 ^22^ | Union | Both | Adult | Professional | Not reported | United Kingdom | Prospective | Match | Time-loss | All injuries |
| Chéradame 2024 ^23^ | Union | Male | Adult | Professional | 2017-2020 | France | Retrospective | Both | Time-loss | All injuries |
| Cosgrave 2019 ^24^ | Union | Male | Adult | Professional | 2016-2017 | Ireland | Retrospective | Match | Time-loss | Head injuries |
| Cruz-Ferreira 2018 ^25^ | Union | Male | Both | Amateur | 2014-2015 | Portugal | Prospective | Match | Time-loss | All injuries |
| Evans 2022 ^26^ | Union | Male | Adult | Semi-professional | 2016-2020 | Wales | Prospective | Both | Medical-attention | All injuries |
| Farley 2022 ^27^ | Union | Male | Adult | Professional | not reported | England | Prospective | Match | Clinical diagnosis | Concussions |
| Fuller 2007 ^28^ | Union | Male | Adult | Professional | 2002-2004 | England | Prospective | Both | Time-loss | Spinal injuries |
| Fuller 2008 ^29^ | Union | Male | Adult | Professional | 2007 | International | Prospective | Both | Time-loss | All injuries |
| Fuller 2010 ^30^ | Seven | Male | Adult | Professional | 2008-2009 | International | Prospective | Match | Time-loss | All injuries |
| Fuller 2011^31^ | Union | Male | Adult | Professional | 2008-2010 | International | Prospective | Match | Time-loss | All injuries |
| Fuller 2013 ^32^ | Union | Male | Adult | Professional | 2011 | International | Prospective | Both | Time-loss | All injuries |
| Fuller 2016 ^33^ | Seven | Male | Adult | Professional | 2008-2015 | International | Prospective | Match | Time-loss | All injuries |
| Fuller 2017a ^34^ | Union | Male | Adult | Professional | 2015 | International | Prospective | Both | Time-loss | All injuries |
| Fuller 2017b ^35^ | Union | Male | Adult | Professional | 2002-2011 | United Kingdom | Prospective | Match | Time-loss | All injuries |
| Fuller 2018 ^36^ | Union | Male | Adult | Professional | 2008-2016 | International | Prospective | Match | Time-loss | All injuries |
| Fuller 2020a ^37^ | Union | Male | Adult | Professional | 2019 | International | Prospective | Both | Time-loss | All injuries |
| Fuller 2020b ^38^ | Seven | Male | Adult | Professional | 2008-2019 | International | Prospective | Match | Time-loss | All injuries |
| Fuller 2021 ^39^ | Seven | Female | Adult | Professional | 2008-2016 | International | Prospective | Match | Time-loss | All injuries |
| Gabbett 2003 ^40^ | League | Male | Adult | Semi-professional | 2000-2001 | Australia | Prospective | Both | Medical-attention | All injuries |
| Gissane 2003 ^41^ | League | Male | Adult | Professional | 1990-2000 | England | Prospective | Both | Time-loss | All injuries |
| Hancock 2024 ^42^ | Union | Male | Youth | Amateur | 2017-2020 | England | Prospective | Match | Time-loss | All injuries |
| Headey 2007 ^43^ | Union | Male | Adult | Professional | 2002-2004 | England | Prospective | Both | Time-loss | Shoulder injuries |
| Kemp 2008 ^44^ | Union | Male | Adult | Professional | 2002-2006 | England | Prospective | Both | Time-loss | Head injuries |
| King 2009 ^45^ | League | Male | Adult | Amateur | 2006-2008 | New Zealand | Prospective | Match | All pain | All injuries |
| King 2021 ^46^ | Union | Female | Adult | Amateur | 2018-2019 | New Zealand | Prospective | Training | Time-loss | All injuries |
| Leahy 2023 ^47^ | Union | Male | Youth | Amateur | 2018-2020 | Ireland | Prospective | Match | Medical-attention | All injuries |
| LopesNPS 2024 ^48^ | Union | Male | Adult | Professional | 2022-2023 | Portugal | Prospective | Match | Time-loss | All injuries |
| LopezVJr 2012 ^49^ | Seven | Both | Adult | Amateur | 2010 | USA | Prospective | Match | Medical-attention | All injuries |
| LopezVJr 2020 ^50^ | Seven | Both | Youth | Amateur | 2010-2014 | USA | Prospective | Match | Medical-attention | All injuries |
| Ma 2016 ^51^ | Seven | Female | Both | Both | 2010-2013 | USA | Prospective | Match | Medical-attention | All injuries |
| McFie 2016 ^52^ | Union | Male | Youth | Amateur | 2011-2014 | South-Africa | Prospective | Match | Medical-attention | All injuries |
| Muma 2012 ^53^ | Union | Male | Adult | Professional | 2010 | Kenya | Prospective | Match | Time-loss | All injuries |
| Murias-Lozano 2022 ^54^ | Union | Male | Adult | Amateur | 2018-2019 | Spain | Prospective | Both | Time-loss | All injuries |
| Murray-Smith 2023 ^55^ | Union | Male | Youth | Amateur | 2018-2021 | Australia | Prospective | Both | Medical-attention | All injuries |
| Pearce 2011 ^56^ | Union | Male | Adult | Professional | 2002-2007 | England | Prospective | Both | Time-loss | Foot injuries |
| Schick 2008 ^57^ | Union | Female | Adult | Professional | 2006 | International | Prospective | Both | Time-loss | All injuries |
| Schwellnus 2014 ^58^ | Union | Male | Adult | Professional | 2012 | South-Africa | Prospective | Both | Time-loss | All injuries |
| Schwellnus 2019 ^59^ | Union | Male | Adult | Professional | 2012-2016 | South-Africa | Prospective | Both | Time-loss | All injuries |
| Starling 2023 ^60^ | Union | Female | Adult | Semi-professional | 2011-2020 | United Kingdom | Prospective | Match | Time-loss | All injuries |
| Stephenson 1996 ^61^ | League | Male | Adult | Professional | 1990-1994 | England | Prospective | Both | Time-loss | All injuries |
| Swain 2010 ^62^ | Union | Male | Adult | Amateur | 2006-2007 | Australia | Prospective | Both | Time-loss | Neck injuries |
| Swain 2016 ^63^ | Union | Male | Adult | Amateur | 2012 | Australia | Prospective | Match | Time-loss | All injuries |
| Tee 2019 ^64^ | League | Male | Youth | Amateur | 2017 | United Kingdom | Prospective | Match | Time-loss | All injuries |
| Tondelli 2023 ^65^ | Union | Male | Adult | Amateur | 2019-2021 | Argentina | Prospective | Both | Medical-attention | All injuries |
| Whitehouse 2016 ^66^ | Union | Male | Adult | Professional | 2014 | Australia | Prospective | Match | Time-loss | All injuries |

Supplementary table 2: Risk of Bias (ROB) assessment for the included studies.

| Study | Was the sample frame appropriate to address the target population? | Were study participants sampled in an appropriate way? | Was the sample size adequate? | Were the study subjects and the setting described in detail? | Was the data analysis conducted with sufficient coverage of the identified sample? | Were valid methods used for the identification of the condition? | Was the condition measured in a standard, reliable way for all participants? | Was there appropriate statistical analysis? | Was the response rate adequate, and if not, was the low response rate managed appropriately? | Quality |
| --- | --- | --- | --- | --- | --- | --- | --- | --- | --- | --- |
| Bailey 2023 | Yes | Yes | No | No | Yes | Yes | Yes | Yes | Yes | High |
| Bitchell 2020 | Yes | Yes | Yes | Yes | Yes | Yes | Yes | Yes | Yes | High |
| Brooks 2005 | Yes | Yes | Yes | Yes | Yes | Yes | No | Yes | Yes | High |
| Brooks 2005 | Yes | Yes | No | Yes | Yes | Yes | Yes | Yes | Yes | High |
| Brooks 2011 | Yes | Yes | Yes | No | Yes | Yes | No | Yes | Yes | High |
| Chéradame 2024 | Yes | Yes | No | No | Yes | Yes | No | Yes | Yes | Medium |
| Cosgrave 2019 | Yes | Yes | No | No | Yes | Yes | Yes | Yes | Yes | High |
| Cruz-Ferreira 2018 | Yes | Yes | No | Yes | Yes | Yes | No | Yes | Yes | High |
| Evans 2022 | Yes | Yes | No | Yes | Yes | Yes | Yes | Yes | Yes | High |
| Farley 2022 | Yes | Yes | No | Yes | No | Yes | Yes | Yes | Yes | High |
| Fuller 2007 | Yes | Yes | Yes | No | Yes | Yes | Yes | Yes | Yes | High |
| Fuller 2008 | Yes | Yes | Yes | Yes | Yes | Yes | Yes | Yes | Yes | High |
| Fuller 2010 | Yes | Yes | No | Yes | Yes | Yes | No | Yes | Yes | High |
| Fuller 2011 | Yes | No | Yes | Yes | Yes | Yes | Yes | Yes | Yes | High |
| Fuller 2013 | Yes | Yes | Yes | Yes | Yes | Yes | No | Yes | Yes | High |
| Fuller 2016 | Yes | Yes | Yes | Yes | Yes | Yes | No | Yes | Yes | High |
| Fuller 2017a | Yes | Yes | No | Yes | Yes | Yes | No | Yes | Yes | High |
| Fuller 2017b | Yes | Yes | Yes | No | Yes | Yes | Yes | Yes | Yes | High |
| Fuller 2018 | Yes | Yes | Yes | Yes | Yes | Yes | Yes | Yes | Yes | High |
| Fuller 2020 | Yes | Yes | Yes | No | Yes | Yes | No | Yes | Yes | High |
| Fuller 2020 | Yes | Yes | Yes | Yes | Yes | Yes | Yes | Yes | Yes | High |
| Fuller 2021 | Yes | Yes | Yes | Yes | Yes | Yes | Yes | Yes | Yes | High |
| Gabbett 2003 | No | Yes | Yes | No | Yes | Yes | Yes | Yes | Yes | High |
| Gissane 2003 | No | Yes | No | No | Yes | Yes | Yes | Yes | Yes | Medium |
| Hancock 2024 | Yes | Yes | No | No | Yes | Yes | Yes | Yes | Yes | High |
| Headey 2007 | Yes | Yes | Yes | No | Yes | Yes | No | Yes | Yes | High |
| Kemp 2008 | Yes | Yes | Yes | No | Yes | Yes | Yes | Yes | Yes | High |
| King 2009 | No | Yes | No | Yes | Yes | No | Yes | Yes | Yes | Medium |
| King 2021 | No | Yes | No | Yes | Yes | No | No | Yes | Yes | Medium |
| Leahy 2023 | Yes | Yes | Yes | No | Yes | Yes | No | Yes | Yes | High |
| LopesNPS 2024 | Yes | Yes | No | No | No | Yes | Yes | Yes | Yes | Medium |
| LopezVJr 2012 | Yes | Yes | No | No | Yes | Yes | Yes | Yes | Yes | High |
| LopezVJr 2020 | Yes | Yes | No | Yes | Yes | Yes | Yes | Yes | Yes | High |
| Ma 2016 | Yes | Yes | No | Yes | Yes | Yes | Yes | Yes | Yes | High |
| McFie 2016 | Yes | Yes | Yes | Yes | Yes | Yes | Yes | Yes | Yes | High |
| Muma 2012 | Yes | Yes | No | Yes | Yes | Yes | Yes | Yes | Yes | High |
| Murias-Lozano 2022 | Yes | Yes | Yes | Yes | Yes | Yes | Yes | Yes | Yes | High |
| Murray-Smith 2023 | Yes | Yes | Yes | Yes | Yes | Yes | Yes | Yes | Yes | High |
| Pearce 2011 | Yes | Yes | Yes | No | Yes | Yes | Yes | Yes | Yes | High |
| Schick 2008 | Yes | Yes | No | Yes | Yes | No | Yes | Yes | Yes | High |
| Schwellnus 2014 | Yes | Yes | Yes | Yes | Yes | Yes | Yes | Yes | Yes | High |
| Schwellnus 2019 | Yes | Yes | Yes | No | Yes | Yes | Yes | Yes | Yes | High |
| Starling 2023 | Yes | Yes | Yes | No | Yes | Yes | Yes | Yes | Yes | High |
| Stephenson 1996 | No | Yes | No | No | Yes | No | Yes | Yes | Yes | Medium |
| Swain 2010 | No | Yes | Yes | No | Yes | Yes | Yes | Yes | Yes | High |
| Swain 2016 | No | Yes | No | Yes | Yes | Yes | Yes | Yes | Yes | High |
| Tee 2019 | Yes | Yes | No | Yes | Yes | Yes | Yes | Yes | Yes | High |
| Tondelli 2023 | No | Yes | No | No | Yes | Yes | Yes | No | Yes | Medium |
| Whitehouse 2016 | Yes | Yes | No | No | Yes | Yes | Yes | No | Yes | Medium |

Supplementary table 3: Anthropometric data on the study participants. The SI units (cm for length, kg for mass) were used.

| **Study** | **Backs (n)** | **Age mean (sd)** | **Height mean (sd)** | **Mass mean (sd)** | **Forwards (n)** | **Age mean (sd)** | **Height mean (sd)** | **Mass mean (sd)** | **Height difference*** | **Mass difference*** |
| --- | --- | --- | --- | --- | --- | --- | --- | --- | --- | --- |
| Bailey 2023 (male) | 25 | 26,6 (3,1) | - | 92,2 (6,8) | 35 | 26,3 (3,6) | - | 113,4 (6,8) | - | 21,20 |
| Bailey 2023 (female) | 16 | 24,7 (3,0) | - | 68,3 (6,6) | 21 | 24,6 (4,4) | - | 82 (9,3) | - | 13,70 |
| Bitchell 2020 | - | - | 183,9 (5,9) | 92,5 (8,0) | - | - | 189,4 (6,9) | 110,8 (8,7) | 5,50 | 18,30 |
| Brooks 2005a | 223 | 24,8 (3,9) | 181,3 (5,5) | 89,4 (6,7) | 269 | 25,9 (4,3) | 188,2 (7,5) | 108,4 (8,2) | 6,90 | 19,00 |
| Brooks 2005b | 31 | - | 181,3 (5,0) | 88,4 (5,4) | 32 | - | 188,0 (8,0) | 106,4 (8,7) | 0,07 | 18,00 |
| Chéradame 2024 | 30 | 29,2 (5,4) | 183,5 (5,9) | 91,2 (7,8) | 42 | 27,1 (5,2) | 188,4 (7,8) | 111,8 (9,1) | 4,90 | 20,60 |
| Evans 2022 | 28 | 25,8 (5,1) | 181,2 (4,9) | 93,3 (5,1) | 41 | 25,5 (4,0) | 184,3 (6,4) | 106,1 (6,9) | 3,10 | 12,80 |
| Fuller 2008 | 284 | 26,9 (3,5) | 182,3 (6,2) | 91,9 (8,2) | 342 | 28,1 (3,4) | 189,0 (7,3) | 110,8 (8,5) | 6,70 | 18,90 |
| Fuller 2010 | 162 | 22,8 (3,1) | 180,1 (6,4) | 86,0 (7,8) | 88 | 23,8 (2,9) | 187,5 (5,8) | 97,7 (7,2) | 7,40 | 11,70 |
| Fuller 2011 | 428 | 19,0 (0,6) | 181,3 (6,0) | 86,5 (8,4) | 513 | 19,0 (0,6) | 187,2 (7,5) | 103,9 (9,7) | 5,90 | 17,40 |
| Fuller 2013 | 281 | 26,7 (3,5) | 182,7 (5,9) | 92,8 (8,2) | 334 | 27,9 (3,6) | 189,2 (7,0) | 111,5 (7,4) | 6,50 | 18,70 |
| Fuller 2016 | 1092 | 23,2 (3,3) | 180,4 (6,3) | 86,4 (7,5) | 724 | 24,2 (3,6) | 187,0 (5,6) | 96,9 (6,7) | 6,60 | 10,50 |
| Fuller 2017a | 279 | 28,2 (3,8) | 188,5 (7,1) | 93,0 (8,9) | 360 | 28,2 (3,8) | 188,5 (7,11 | 112,6 (9,0) | 0,00 | 19,60 |
| Fuller 2018 | 1748 | 19,1 (0,85) | 180,9 (6,2) | 86,7 (8,3) | 2174 | 19,1 (0,8) | 187,0 (7,3) | 104,9 (10,1) | 6,10 | 18,20 |
| Fuller 2020a | 284 | 27,3 (3,5) | 182,6 (6,0) | 91,2 (9,1) | 362 | 27,3 (3,8) | 188,3 (7,0) | 111,8 (9,2) | 5,70 | 20,60 |
| Fuller 2020b | 3242 | 23,4 (3,4) | 180,5 (6,3) | 86,8 (7,6) | 1289 | 24,4 (3,7) | 187,2 (5,3) | 96,8 (6,6) | 6,70 | 10,00 |
| Fuller 2021 | 882 | 23,7 (3,8) | 167,2 (5,3) | 65,6 (5,6) | 680 | 24,5 (3,8) | 170,7 (5,6) | 71,2 (6,3) | 3,50 | 5,60 |
| King 2009 | 41 | 25,5 (4,2) | 177,0 (7,0) | 81,4 (11,3) | 53 | 26,4 (3,9) | 180,0 (5,0) | 99,0 (19,4) | 3,0 | 17,6 |
| LopezVJr 2020 | 92 | 17,3 (4,6) | 174,3 (8,1) | 80,9 (11,0) | 56 | 17,3 (4,6) | 173,2 (6,8) | 72,4 (9,7) | -1,10 | -8,50 |
| Murias-Lozano 2022 | 114 | 24,3 (3,8) | 178,5 (5,9) | 83,2 (8,8) | 144 | 26,2 (5,0) | 184,1 (7,1) | 102,5 (11,6) | 5,60 | 19,30 |
| Schick 2008 | - | 27,0 (4,8) | 165,6 (5,9) | 68,0 (7,5) | - | 29,0 (4,6) | 172 (6,7) | 80,2 (10,1) | 6,40 | 12,20 |
| Schwellnus 2014 | 67 | 25,2 (3,6) | 183 (6,0) | 90,5 (6,3) | 85 | 24,9 (3,3) | 189,0 (7,0) | 110 (7,9) | 6,00 | 19,50 |
| Starling 2023 | - | 25,0 (2,0) | 155 (9,0) | 68,0 (2,0) | - | 26 (2,0) | 168,0 (10,0) | 77,0 (2,0) | 13,00 | 9,00 |
| Swain 2016 | - | - | 182,8 (6,3) | 86 (10,5) | - | - | 184,1 (7,3) | 92,8 (11,3) | 1,30 | 6,80 |
| Tondelli 2023 | 74 | 25,3 (6,1) | - | - | 100 | 24,7 (4,6) | - | - | - | - |
| Whitehouse 2016 | 82 | 24,5 (2,7) | 184,94 (5,4) | 91,4 (7,1) | 98 | 25,2 (3,4) | 189,3 (7,4) | 111,2 (7,1) | 4,36 | 19,8 |

* Forwards – backs. Sd = standard deviation
